# Essay on the Sources and Mode of Propagation of the Continued Fevers of Great Britain and Ireland

**Published:** 1841-01

**Authors:** William Davidson

**Affiliations:** Senior Physician to the Glasgow Royal Infirmary, Lecturer on Materia Medica, Member of the Faculty of Physicians and Surgeons of Glasgow, &c.


### APPENDIX.

### THACKERAY PRIZE ESSAY.

*Essay on the Sources and Mode of Propagation of the Continued Fevers of Great Britain and Ireland.* By William Davidson, m.d., Senior Physician to the Glasgow Royal Infirmary, Lecturer on Materia Medica, Member of the Faculty of Physicians and Surgeons of Glasgow, &c.

### Chap. I. On the Sources of Continued Fevers.

Numerous kinds of continued fevers have been described by authors; but many of these have been found, on investigation, only particular varieties, in place of being distinct species. This has been particularly the case with typhus, the most prevalent kind of continued fever in this country; and it may be accounted for, from its numerous and diversified complications giving rise to various and multiform symptoms. The pathology of typhus, however, of late years has been considerably advanced; and it is now established, that this disease may be either simple or complicated, with organic affections of one or all of the different cavities of the body.

We shall not, at present, enter into any discussion respecting the various kinds of continued fever that are to be met with in the United Kingdom; but as perspicuity in arrangement requires some classification, we shall adopt the following, reserving the illustrations upon which this classification is founded for a future part of the essay:

1. Typhus.
2. Febricula or Simple Fever.
3. Gastric or Intestinal Fever.

These affections seem to be distinct species of disease, differing in their symptoms, causes, and laws; and are generally treated in private practice and in hospitals as continued fevers. To this list may, perhaps, be added bronchitis which, although an inflammatory affection, is more frequently confounded with them than any other disease.

### Sect. I. Sources of Typhus.

It appears to us quite unnecessary here to describe what is understood by typhus fever; as we assent to the general correctness of our standard authors upon this subject; and as some of their descriptions will be quoted in another part of this essay. At the same time it may be remarked that typhus possesses an advantage, over the other forms of continued fever, in having a distinctive characteristic, viz., the eruption, which is present in none of the others, and which is now almost universally acknowledged as decisive of its existence. It must, however, be admitted that typhus can and does occur, without its characteristic eruption; but it is equally certain that the large majority of patients who have decidedly the general typhoid symptoms, are more or less spotted with this efflorescence. It is therefore the sources of typhus as generally so characterized, which we mean to trace in this section.

There is considerable diversity of opinion amongst British physicians respecting the causes of continued fevers; but certainly the majority of authors have adopted the belief that typhus is propagated by contagion. The opinion of the majority appears to be supported by the facts connected with the progression of the disease; it shall therefore be our object to establish this point. It is not intended, however, to enter into any speculations respecting the primordial source of the contagion of typhus; for the sources from which it, as well as that of other contagious fevers, originated, is involved in absolute obscurity; and though we could trace them to the most remote era in antiquity, the same difficulty would be encountered. Some authors, apparently to get rid of this difficulty and to account for the occurrence of typhus where no contagion could be traced, have adopted the opinion that it may be generated by common causes, such as impure air, filth, &c., and be afterwards capable of propagation by contagion.* The argument of analogy is directly opposed to this belief; for if, in nature, there be no exception to the law, that two causes are never required to produce precisely the same effect, it will follow, that whatever cause can be best reconciled with the phenomena of typhus, must be considered the true source of the disease. But, in order to apply this principle more immediately to the subject, it may be necessary to appeal to the various morbid poisons, the laws of which are known and generally admitted. The first we shall notice are those which are admitted by all writers to be propagated by one cause only; viz. matter, whether ponderable, as the pus contained in a variolous pustule, or imponderable, as the effluvia issuing from a patient labouring under smallpox. Measles, scarlet fever, hooping-cough, are propagated only by the *effluvia* which are generated by the patient; and though the material body, by which it is effected, cannot be collected into vessels, like the various gases, still the proofs, upon which their contagious qualities are based, are as unquestionable as those of smallpox. Almost all the contagious diseases of the skin, such as syphilis, scabies, the yaws, sivvens, &c., furnish examples of propagation by only one cause, viz. contagion. There are no doubt authors who maintain that some cutaneous diseases are generated by filth, &c., such as some of the infectious species of porrigo; but of this there is no proof; and in all probability it is equally unfounded as was a similar hypothesis respecting the origin of scabies. These diseases always retain the same characteristics; the one is not convertible into the other; and no known combination of them can generate a new contagion capable of perpetuating a new disease. A specious objection might be brought forward against the introduction of an analogy from chronic contagious diseases, which are only propa-

* The terms contagion and infection are used synonymously, as indicating the ponderable or imponderable matter, which is generated in a diseased living body, and which is capable of producing the same disease when applied to another.

gated by contact, according to the general belief; and besides are regulated in other respects by different laws, whereas typhus is propagated only by effluvia. It is quite evident that a class of diseases may be recognized by one leading and undeviating law, while they differ in many of their subordinate characters; and yet this peculiar law of similarity between them may be as certain and definite as if they had been united into one family by all their habits. The effluvium which issues from a smallpox patient must be as essentially matter as is the fluid of a variolous pustule; for though the first cannot be collected in a separate form, it must possess one or other of the properties of smallpox matter, else it could not induce the disease; the only difference between them consisting in this, that the contagious matter is fluid in the one case and effluvial in the other. It is necessary that the foregoing observations respecting contagious diseases be kept in view; for upon the analogy between them and typhus we mean to establish an argument, that the latter disease can only be propagated by one cause. If it be true then, that all the contagious fevers known can be propagated only by contagious matter, and by no other cause, however much their contagious qualities, their prevalence, and their fatality may be increased by other causes, it must follow from the law of analogy that if typhus can be proved to be contagious, it must also be propagated only by one cause, viz. contagion. We shall, therefore, endeavour to prove this point; and, in the outset, it may be stated, that we do not mean to fatigue the reader by stories about fomites and persons who have carried the contagion about them for months or years, nor to hunt out a particular individual who has conveyed it from one town to another. In place of accumulating evidence of this kind, which although sometimes very conclusive, is in other cases somewhat questionable; we shall select a few facts from the history of our British and Irish hospitals, which, we trust, will be sufficient for our purpose; for if it can be established from these documents that the disease was contagious in all the large hospitals of Britain and Ireland, then it must be more or less so in every other place. In selecting the facts, we shall adduce the most conclusive instances, such as where the whole or almost the whole of the attendants of the patients affected with typhus were infected; for were the whole body of evidence existing on this subject accumulated in this essay, the argument would be encumbered, and the proofs perhaps rendered less convincing. Those who deny the existence of typhous contagion may assert that this is unfair, and that those hospitals also should be brought forward, where the medical and other attendants were rarely affected. As we shall, however, notice this and other objections elsewhere, it need not be farther alluded to here. Drs. Barker and Cheyne in their admirable report of the fever which prevailed in Ireland during the years 1817-18-19, state that "in the hospitals of the house of Industry of Dublin, no clinical clerk or apothecary escaped an attack of the disease; and on the 20th January, 1819, it was reported to Government that five of the medical attendants of the house of Industry were at that time lying ill of the disease. In the city of Cork nine physicians in attendance either on dispensaries or fever hospitals were attacked; every medical attendant at the South Fever Asylum in that city suffered. At the hospitals of the House of Industry, 170 persons were employed in different offices of attendance on fever patients, and from this part of the establishment were recorded 198 cases of fever." In Dr. Crampton's medical report of the department of Steevens' Hospital it is observed "that, with the exception of Dr. Harvey and himself, all those concerned in attendance on the patients caught the disease; none of the nurses, none of the porters, barbers, or those occupied in handling, washing, or tending on the sick escaped, and many of them had relapses and recurrences of fever."*

Dr. Bracken in his report of the Fever Hospital of Waterford for 1818, states that "there were twenty-seven attacks and relapses of fever among the nurses, servants, and porters, whose number fluctuated according to the demand for them, but who, on an average, may have been about twenty-two during the year." He farther states that "the present year 1819 bears a close resemblance to the last, in respect to the nurses and servants being attacked with fever, eighteen of the former having suffered under the disease; seven of them once, three twice, and one three times." The apothecary, who had not been long in the hospital, caught fever and relapsed twice. During his illness, a young man, who performed part of his duties, was attacked after a short attendance. A temporary apothecary was then engaged for a few weeks; but he had not been many days in his new employment when he also contracted a fever."†

Drs. Barker and Cheyne remark that clergymen who visited typhus patients in Ireland during the epidemic were also observed to suffer in a very remarkable degree; and they quote the following passage from Dr. Stokes' Essay on Contagion, which was published at a time when the fever had made little progress in Dublin: "The deaths from fever recorded in Saunders's News-letter, from August 1st to December 12th following, are sixty-four, and of these nineteen are of clergymen of some of the different persuasions, or of medical men of different descriptions, which appear greater than the proportion which these two classes bear to the whole of those whose deaths we may suppose were mentioned in that manner."‡ Dr. Tweedie in his Clinical Illustrations of Fever for 1828-29, observes that "the London Fever Hospital is placed in an open space, situated in the vicinity of the metropolis, close to the Smallpox Hospital. Both these establishments stand in the centre of a large field, where the production of malaria is extremely improbable. I can state from the most authentic sources, that every physician who has been connected with it, with one exception (the late Dr. Bateman), has been attacked with fever during his attendance, and that three out of eight physicians have died. The resident medical officers, matrons, porters, laundresses, and domestic servants not connected with the wards, and every female who has ever performed the duties of a nurse, have one and all invariably been the subjects of fever; and to show that the disease may be engendered by fomites in clothing, the laundresses, whose duty it is to wash the patients' clothes, are so invariably and frequently attacked with fever, that few women will undertake this loathsome and

* Barker and Cheyne's Report of the Fever in Ireland, vol. i. p. 135.

† Ibid., vol. i. p. 276.

‡ Ibid., vol. i. p. 138.

frequently disgusting duty. Last summer a most convincing illustration of contagion occurred. The present resident medical officer was attacked with fever, and it was necessary, in consequence, to appoint some one to perform his duties during his illness. The first person who officiated for him resided constantly in the house during the day, but took the precaution of sleeping at home. He was, of course, very much exposed in the wards in the performance of his duties. These, however, were soon interrupted by an attack of fever, which confined him for a considerable time. The duties were then undertaken by a medical pupil, who had completed his education, and entered the hospital in the most robust health. He had been taught, and did implicitly believe, in the non-contagious nature of fever, and ridiculed the idea of any personal danger from residing in the hospital. He performed the duty of house-surgeon for ten days only, when symptoms of a severe fever appeared."* Dr. Tweedie also adduces some important facts connected with the fever which prevailed in Edinburgh during the year 1817, which are the following: Owing to the prevalence of fever at Edinburgh in 1817, it was necessary to apply to government to permit Queensbury House to be employed as a fever hospital: "In the immediate neighbourhood of this extensive building fever was decidedly less prevalent than in any other quarter of the town. All those, however, who resided in the hospital, including the resident house-surgeon, clerks, apothecary, and nurses, were successively attacked." The following is Professor Alison's report on this subject. "When Queensbury House was formerly occupied by fever patients, every resident clerk and every nurse in the house were successively affected with the disease; and since it was reopened in December last (1826), the resident physician, two of the clerks (who have not been resident, but have been several hours in the day in the house), the apothecary, several servants, and all the nurses except two, in all above forty individuals, who had necessarily close intercourse with the sick there, have had fever. If this be the effect of a malaria, it must be a very virulent and effective one, and it is reasonable to expect that some record of similar visitations in the former history of the building would be found. But Queensbury House has existed for about a century; it was long occupied as a private dwelling-house by the noble family of that name; afterwards it was occupied by a number of families, and afterwards as a soldiers' barrack; and yet no record can be found of its having been, during these changes, the seat of an epidemic fever. If a malaria has existed, therefore, in that house, it must, on both occasions, have sprung up exclusively at the times when fever patients were removed thither and lasted only during their stay. During the present epidemic (1827-28, as well as that of 1817-19), many of the clerks and nurses employed in the Royal Infirmary have taken fever. Since November last, six of the clerks employed in the clinical wards only, four of those employed in the ordinary wards, and twenty-five nurses or servants have taken fever. All these persons had necessarily frequent and close intercourse with the fever patients in the house, having been employed more or less constantly in the fever

* Tweedie's Clinical Illustrations of Fever, p. 87.

wards, excepting only four of the servants. Of these four, two had been employed in the laundry, where the linen from the fever wards was washed. One was a porter employed at the gate, who would, of course, have communication with the fever patients at their entrance and dismissal, as well as with their relations coming to visit them; and one was a nurse employed in the servants' ward, but who was in the habit of visiting the fever wards." He adds further: "No one of the nurses, whose duty has confined them to the medical or surgical wards, where no fever patients were admitted, has taken fever, with the single exception of the woman in the servants' ward above mentioned; and of the numerous patients in these ordinary wards, the only one who has taken fever, within my knowledge, during the present year, was a patient in the men's general clinical ward, who lay in the bed next the door that communicates with the clinical ward."* Dr. West, in his account of the cases of typhus exanthematicus that occurred in St. Bartholomew's Hospital in 1837-38, states that "since last summer, eleven gentlemen who were in the habit of frequenting the hospital have been attacked by the fever, to which three have fallen victims; sixteen nurses and twenty-one patients admitted for other affections, have likewise suffered from the disease, which terminated fatally in ten instances, and I do not doubt but that many similar cases occurred which did not come under my notice."† Dr. Roupell, also, gives similar testimony in reference to St. Bartholomew's Hospital, and states that "amongst the nurses, in attendance upon the sick, in that establishment, infection was almost universal."‡ In the Glasgow Fever Hospital, which is capable of containing 220 patients, during the last six or seven years almost every clerk and nurse of that establishment have caught fever while acting in the wards, unless they had previously laboured under the disease. On the other hand, the nurses connected with the medical and surgical wards, in the adjoining building, have almost uniformly escaped. Occasionally a case has appeared in the medical and surgical wards; but this fact ought to be coupled with the statement, that now and then typhus cases are sent, by mistake, into the medical wards, and cases of bed-sores, gangrene of the feet, &c. are transmitted from the fever hospital to the surgical wards. Dr. Cowan states that "All the gentlemen who have acted as clerks in the fever hospital for many years past have been attacked with fever, unless they had it previously to their election. During last year, twenty-seven of the nurses of the establishment were seized with fever, and five of them died, several of the students have been affected. One gentleman who acted as apothecary died in the house; and if I have escaped, it must be attributed either to being past the period of life at which fever usually takes place, or to my being secured by having had two dangerous attacks at an earlier period of my career, when acting as physician's clerk in the infirmary, during the epidemic of 1816-17-18."§

* Tweedie's Clinical Illustrations of Fever. Edinb. Med. and Surg. Journal, vol. xxviii. p. 238.

† Ibid., July, 1838, p. 143.

‡ Roupell on Typhus.

§ Cowan's Vital Statistics, p. 26.

Dr. Mateer gives a table of 9,588 cases, which were admitted into the Belfast Fever Hospital, from 1818 to 1835, showing the number of patients who had any communication with affected persons, either by residence in the same house, or by belonging to the same family. He draws the following conclusion from the table: "It thus appears that the number of families where contagion is traceable is 1,856, that the total number of persons belonging to them is 7,246, making an average of nearly four individuals to each family; and that the single cases, where the disease seemed to have arisen from other sources, amount only to 2342."*

This assemblage of facts has been drawn from the large hospitals in England, Scotland, and Ireland, and the observations have been made during various years and during different epidemics by gentlemen of the highest talents and respectability; their authenticity cannot, therefore, for a moment be questioned. The simple relation of these facts would, we think, with the majority of men, produce conviction that fever was at least contagious in these hospitals, provided the mind was not preoccupied with an opposite theory; but a few observations will tend to produce a proper estimation of this testimony. It is quite obvious, that where a much larger proportion of persons is affected with any particular disease, in any particular place, than occurs amongst the general community, or in any particular grade of society, there must be some local cause for that increased ratio. This has, manifestly, been the result in the fever hospitals that have been enumerated; for in all, a very large majority of the attendants, and in some the whole of them, were affected with fever. Now, no one will contend, that even amongst the lower classes (who generally suffer from fever to the greatest amount), such a proportion has ever been maintained, even in our most severe typhoid epidemics; but if the number of hospital clerks be taken and compared with the unaffected number, in the particular grade of society to which they belong, such an attempt would be ridiculous; for the united testimony of the hospital physicians of England, Scotland, and Ireland (whose statements we have already quoted) amounts to this, that almost every clerk of a fever hospital has laboured under fever during some period of his attendance upon it. It may be contended, in answer to this argument, that the atmosphere of the hospitals was contaminated by the exhalations arising from the number of patients and the want of proper ventilation; but the same process of atmospheric deterioration ought to take place, in the medical and surgical wards, if they be equally filled, which is generally the case; and in the latter wards there are often, in addition to the ordinary exhalations, the effluvia arising from wounds, ulcers, &c., yet typhus rarely, and only in sporadic cases, springs up there.

The opponents of contagion, however, endeavour to explain the prevalence of fever among hospital attendants by the hypothesis, that the same cause that produced it in the filthy, ill-ventilated houses of the lower classes is in existence in these institutions; viz., a peculiar ma-

* Dublin Journal of Medical Science, vol. x. p. 35.

laria generated, chiefly, in large towns. If this hypothesis were true, it would follow, as a necessary consequence, that the other parts of the building, being similarly situated, would be subjected to the same malarious effluvia, and hence its inmates would be affected with the same kind of disease; but this has never occurred in any of the large hospitals already alluded to, nor in any other, so far as we are aware, where patients affected with typhus are kept exclusively in one place. Again, it may be asserted by the non-contagionist, when driven to the last extremity, that though malarious effluvium be not generated in an hospital, it may be carried there by the clothes of the patients, and the attendants may be infected by coming into contact with them. The analogy of malarious diseases is in opposition to this belief; for it is not found that a patient labouring under ague infects any person who has not been in the malarious district; neither, according to the general belief, does a patient labouring under yellow fever, when removed from the quarter where he caught the disease, excite contagion in the vicinity of his new residence. But as this supposed typhoid malaria may be assumed to possess something *sui generis*, an argument stronger than analogy can be adduced, viz., the impossibility of carrying any principle of that kind into the wards. It is the practice, in many of the large fever hospitals, to remove the clothes of the patients, to bathe them, shave their heads, and give them clean linen, before they are sent into the wards. This plan is adopted in the Glasgow Fever Hospital, and the following is one of the regulations of the Waterford Hospital.

Dr. Bracken states, that "according to one of the regulations of the hospital, every patient has his hair closely cut at the time of his admission; he is also well washed with warm water and soap, and supplied with linen before he enters the sick ward."*

### Sect. II. On the Analogy of Typhus to Exanthematous Fevers.

Having discussed the most important and specious hypotheses which have been brought forward to explain the general and ultra-proportionate prevalence of fever among the attendants of the sick in large hospitals, independent of the operation of contagion, we shall take notice of the general objections to the doctrine of contagion in typhus fever; and these may be comprehended in the statement, that it is not characterized by the laws of other contagious fevers. Before entering upon this part of the subject it may be remarked, that more importance is generally attached to this argument than it merits; for though, in the absence of facts, analogy is the most conclusive process of reasoning that can be employed, yet, undoubtedly, when facts are opposed to the application of this principle in any individual case, the facts have the preponderance over the analogy. And, though we were unable to prove that typhus was analogous, in its leading characteristics, to the contagious exanthemata, yet if it be admitted that there is no theory which can explain satisfactorily the facts regarding the prevalence of the disease in

* Barker and Cheyne on Fever, vol. i. p. 259.

hospital attendants, except that of contagion, the case would be conclusively determined, even in opposition to the analogy, and would be set down either as an exception, or as one of a new series of contagious diseases. It fortunately happens, however, that the law of analogy will be little violated by comparing typhus with the contagious fevers, for in its leading characteristics it resembles them pretty closely; at the same time it ought to be observed, that typhus fever has only, of late years, been examined with sufficient care, as to many points, connected with its history, laws, and pathology, and that it labours under the disadvantage of being frequently confounded with other continued fevers, to which, in its early features, it bears an intimate resemblance; so that the same certainty of analogical conclusion cannot be expected, as exists among the other exanthematous fevers. We shall endeavour, however, to show, by the facts which shall be quoted, that typhus comes distinctly within the range of their analogy, and that though it is not so regular in its progress, nor so certain in its eruption as smallpox or measles, yet that it differs as little from scarlet fever, in these respects, as the latter differs from smallpox.

The principal laws of the contagious exanthemata are the following:

1. The contagion can be traced in families, hospitals, schools, &c., and those exposed to it are very enerally infected.

2. They only affect persons once during their lives.

3. They are characterized by an eruption, which has a rise, progress, and decline, and the disease cannot be checked in limine.

1. *The contagion of typhus is traceable in hospitals, schools, families, &c.* In determining the contagious nature of any disease, it is not necessary that we should be able to trace every case, or even the majority of a particular amount of cases, to a communication with an infected person, or to exposure to a particular fomites; for this would imply that we could, like an American Indian, discover the trail of a patient and trace him through all the windings of a large city, and, besides, should investigate the history of every individual whom he has passed or rubbed shoulders with in every narrow and dirty alley. Even in smallpox, measles, and scarlet fever, any attempt of this kind to trace the contagion regularly would be fruitless, and for the very same reason. Smallpox was, at one period, believed by some authors to originate in filth, because it was found impossible, in numerous cases, to account for its existence in certain localities upon the principles of contagion. Dr. Adams remarks, that "many children born in London live for several years without receiving the smallpox. In the same neighbourhood a person arrives from the country, and without any apparent intercourse with an infected person is attacked by the disease."*

Independent of the many exposures to infection, which are perfectly unknown and undiscoverable by the patient, it is very difficult to ascertain the facts connected with the ordinary movements of a patient, which, in many cases, can only be elicited by tedious cross-examinations; so that this method of determining the point is liable to many objections, and greatly inferior in conclusiveness to the evidence derived from the

* Adams on Morbid Poisons.

spread of a disease in any large school or hospital; but, certainly, it tends to prove the doctrine of contagion in typhus as much, if not more, than it does in smallpox, &c., as will appear from the following table. The whole of the eruptive cases of typhus, in which this point was investigated, and that were admitted into the Glasgow Fever Hospital from 1st May to 1st November, 1839, are included only in this table, the males and females being classed together.

| | Exposed to Contagion. | Uncertain. | Cold. | Total No. of Cases. |
|---|---|---|---|---|
| Eruptive typhus . . . | 201 | 169 | 53 | 423 |
| Febricula . . . . . | 10 | 28 | 22 | 60 |
| Smallpox . . . . . | 7 | 19 | 1 | 27 |
| Scarlet fever . . . . | ... | 4 | ... | 4 |
| Measles . . . . . | ... | 2 | 1 | 3 |

The number of eruptive cases of typhus admitted into the fever hospital, both in the period included in the above table and also during the previous six months, who have been exposed to contagion, we have always found greater than in those affections not characterized by the exanthema; and it is remarkable that, notwithstanding the most careful enquiries, only 7 cases of smallpox could be traced to contagion out of 27.

Dr. Cowan states, that " of the patients admitted into the fever hospital last year, 472 males and 589 females, forming 47 per cent. of the whole, either ascribed the origin of their disease to contagion, or had been exposed to its influence."*

The propagation of the typhoid contagion is also intimately connected with filth and deficient ventilation; and there are few medical facts better ascertained than the close connexion of pestilence with these circumstances. Dr. Hancock remarks, that "the connexion of plague with filth and impure air, and crowded ill-constructed cities, and with certain seasons and climates and states of the atmosphere, calculated to engender mischief, though not accurately defined, has been so repeatedly observed in different countries as to stand on a far more solid foundation."† Dr. Bateman, in his Historical Survey of the Diseases of London, states that " Dr. Heberden has collected the most ample and satisfactory evidence of the connexion of the plague, and of the malignant contagious fever which generally precedes and accompanies it (if, indeed, they be not modifications of the same disease) with the filth of crowded, ill-ventilated large cities, in all ages and countries." He then quotes Dr. Heberden's remarks: " It has always originated and maintained its head-quarters in the filthiest parts of those cities; as in St. Giles's, in London, in 1665, and in Whitechapel in 1626 and 1636; and in those cities of Europe, which, from natural or political causes, have been backward in adopting the improvements of modern times: the picture of former manners is not exhibited in more lively colours than that

* Cowan's Vital Statistics of Glasgow, p. 26.
† Hancock on Pestilence, p. 224.

of former diseases. The plague visited Denmark in 1764, it raged at Moscow in 1771, and at Cracow still later. The last-mentioned town, Mr. Wraxall says, was not wholly paved till within the last two years, and nothing can be so execrable as the present paving, which scarcely deserves the name. There is not a single lamp in the place; no precautions are used to cleanse the streets, which, of course, become infectious in summer and almost impassable in winter." The following is Erasmus's description of the habits of the English, about two centuries ago: "The floors are commonly of clay, strewed with rushes, which are occasionally renewed, but underneath lies unmolested an ancient collection of beer, grease, fragments of fish, spittle, the excrement of dogs and cats, and everything that is nasty."* Dr. Hancock observes, that most writers on the plague have remarked the exemption of Persia from this disease, and he quotes the following passage from the City Remembrancer: "The Persians, though their country is every year surrounded by the plague, seldom suffer anything by it themselves; they are the most cleanly people in the world, many of them making it a great part of their religion to remove filthiness and nuisances of every kind from all places about their cities and dwellings."† Drs. Barker and Cheyne, in their statement of the circumstances which either preceded or attended the epidemic fever in Ireland during the years 1816 and 1817, make the following remarks, which may be assumed as conclusions drawn from the reports of physicians practising in the various provinces, and from the observation of more than 100,000 cases in general hospitals: "When fever commenced in a poor family, or was introduced by a stranger or lodger, it generally extended to all its members. The poor were the chief sufferers, in consequence of their neglect of cleanliness, particularly with respect to their clothing, and the smallness and crowded state of their apartments; evils, at the time, much increased by the extreme poverty which weighed them down. On the other hand the superior classes, whose circumstances were different, their clothing more frequently changed, their persons more cleanly, their apartments less crowded and better ventilated, and among whom seclusion from the sick was practised, in proportion to their enjoyment of these advantages, generally escaped the disease."‡

Dr. Bateman, after describing the methods to be adopted for promoting cleanliness and sufficient ventilation, remarks: "If these simple measures be steadily pursued, no confinement or accumulation of morbid effluvia can take place under any state of fever; and the air of the apartment may be breathed, and the bed and person of the patient approached and touched with perfect impunity. If this were not the case, indeed, physicians and nurses, especially those employed in fever hospitals, would have little security for their lives. During the fourteen years, in the course of which I have almost daily been in contact with persons labouring under contagious fever, not only myself but all the nurses have thus been preserved from infection, with one excep-

* Bateman on the Diseases of London, p. 18.
† Hancock on Pestilence, p. 287.
‡ Barker and Cheyne on Fever, vol. i. p. 134.

tion, down to the period of the present epidemic." He adds in a note: "It is no disparagement to the system above described that some of the nurses and the matron of the House of Recovery have been infected during the present epidemic, which has kept the wards constantly full. The impossibility of maintaining a free ventilation night and day, during the cold weather, their perpetual exposure, in close contact, to the breath and discharges of the patients, while feeding, moving, or washing them, changing their beds and linen, and even stripping off their infected clothes on admission, might be sufficient to counteract the solitary operation of any general system, however efficacious. But the truth is, that the ventilation of the house has been very imperfect, and even at the command of the nurses and patients; and the injurious consequences of this imperfection have become so manifest, that the subject is now under the consideration of the committee, while this work is in the press."*

Dr. Hancock quotes the following facts, which illustrate very powerfully the influence of ventilation: "In the year 1819, I had occasion to see a very intelligent physician connected with one or two fever hospitals in Dublin, during the epidemic, who assured me he had seen no proof of the existence of contagion in the disease (typhus) as it appeared in those institutions under his care, where very great attention was paid to ventilation, and where the patients were not inconveniently crowded. But soon after this, I saw another physician no less intelligent, who informed me that in the course of about four months, between 200 and and 300 persons were admitted into the Belfast Fever Hospital; and they were frequently so crowded in the wards as nearly to cover the floor with their beds; in which case, although the building is new, airy, and well regulated, the matron, twenty-two nurses, and the apothecary, took the disease; yet it was so mild, that scarcely more than one in fifty died."†

Dr. Prichard relates a striking example of the effects of a good as well as of a deficient ventilation, which occurred in two of the hospitals in Bristol, namely, St. Peter's and the Bristol Infirmary; both of these institutions being under his medical superintendence. "In the former, (St. Peter's,) the medical wards are very small, and it was necessary to place the beds very near to each other, and to put too great a number of patients in a given space. Offensive smells were often perceptible; and it was under these circumstances that the disease was manifestly contagious." In the Bristol Infirmary the wards are lofty and well ventilated. "Here, also, the fever patients were dispersed among invalids of almost every other description. But no instance occurred of the propagation of fever; none of the nurses were attacked, nor were the patients lying in the adjacent beds in any instance infected, though cases of the worst description of typhus gravior were placed promiscuously among the other patients, scarcely two foot of space intervening between the beds."‡

* Bateman on Contagious Fever, p. 154.

† Hancock on Pestilence, p. 339.

‡ Hancock on Pestilence. Prichard's History of the Fever in Bristol, p. 88.

Drs. Barker and Cheyne state that "a remarkable proof was afforded in Sir Patrick Dunn's Hospital of a ward, by the peculiarity of its construction, protecting the attendants upon the sick from the effects of contagion. The ward alluded to is the fever ward for males, which extends the entire breadth of the left wing of the hospital, being sixty-two feet by thirty-eight. It is twenty feet high, and is subdivided by partitions, of the height of nine feet, into six apartments, two of which are thirty-eight feet by sixteen, and the rest are each nine feet square; the latter contains, with great convenience, four beds each, and the former ten; but on occasions of necessity, the square apartments have held five and the oblong twelve beds without inconvenience; the partition walls leave two passages, one leading from the door of the wards across its breadth and another passing in the middle of its length; it is furnished with three large fireplaces, two of which are in the oblong chambers, one on the north and the other in the south side of the ward, and the third opposite the door, at the end of the passage first described; by this door, the fever-ward opens on the staircase which is walled and communicates with the corridors of the basement and underground stories. The greater number of the windows of the ward are sixteen feet from the floor, and in the ceiling are placed two louvres, one toward either end, by means of which and the fire-places a brisk ventilation is kept up. During the late epidemic, when Sir Patrick Dunn's Hospital, by agreement with government, contained 100 patients in fever, the male ward was crowded, containing forty-four patients, yet only one nurse was affected with fever; at the same period, the nurses in attendance on the female patients, who were certainly not so much crowded together, were continually taking the complaint and generally had it with severity."* In addition to the facts which have now been brought forward, it may be stated, without much chance of contradiction, that as there is in almost every large town deficient accommodation for fever patients in an hospital during an epidemic, that over-crowding is an ordinary result, from the anxiety of the directors to relieve the misery of the sick. The Glasgow Fever Hospital is calculated to contain 220 patients; and for nearly two years, namely, during 1836-7, it was generally filled to its maximum, and frequently from ten to twenty additional were accommodated. Now, it is quite obvious, that such a large number of fever patients, all contained in one building, will exhale a prodigious quantity of typhoid effluvium, which must be exceedingly concentrated; and that even the utmost cleanliness and the greatest degree of ventilation consistent with the temperature that ought to be maintained, would scarcely be sufficient for its proper dilution.

Drs. Barker and Cheyne remark, in that portion of their report which has been already quoted, that typhus generally spreads in the families of the lower classes and very rarely in those of the superior ranks. Dr. Cowan states that "the fever was chiefly, nay almost wholly, confined to the labouring classes and to the districts which they inhabited, while among the wealthy and middle classes of society it was comparatively seldom met with, and when it did occur, was not spread by contagion

* Barker and Cheyne on Fever, vol. i. p. 488.

through all the inmates of the family, as was usually the case among the families of the poor, but was confined to a single individual."* These results, as stated by the above-mentioned authors, agree, we are convinced, with those which have been made in almost every other place. This remarkable difference, in the two classes of persons referred to, must be owing chiefly to the wide diversity of circumstances in which they are placed; and approximates very closely to the difference which exists between a crowded and consequently an ill-ventilated hospital, and one which is limited to a small number of patients with thorough ventilation. The lower classes in large cities generally live in dirty ill-ventilated houses, and are often filthy in their persons; while the better ranks live in more airy situations, have larger houses, and are more attentive to cleanliness in their persons and domestic habits; hence the effluvium which issues from a typhus patient, in the first-mentioned situations, cannot be carried off so readily, or diluted to the same extent with atmospheric air as in the second. But it may be said by the opponents of typhoid contagion, that smallpox, measles, and scarlet fever more frequently spread in the families of the better ranks than typhus; and why is ventilation and dilution not effectual in these cases? In answer to this objection, it may be stated, that these three last-mentioned diseases are not equally contagious, and that scarlet fever, particularly when it is not epidemic, is often confined to one person in a family; whereas smallpox, in the majority of instances, affects the greater number of unprotected persons, adults as well as children. M. Rayer states that "scarlatina is contagious, but to a less degree than measles. It affects chiefly children and young persons, more rarely adults. Every individual is not, to the same degree, apt to be affected with scarlatina, and every condition is not equally proper for its development. It attacks females more readily than males; and some individuals, after having been exposed, in vain, during many days to the contagion of this disease, have been seized after the lapse of some time, in consequence of a simple communication with persons who had visited patients affected with this exanthema."†

Dr. Bateman states that adults are not very susceptible of the disease (scarlet fever), and that many medical practitioners who have attended great numbers of patients affected with it have never experienced its effects.‡

Dr. Mason Good observes that "nothing is more common than for a sporadic case of rosalia (scarlatina) to occur in a family without communication of itself to the surrounding children, although no pains may have been taken to keep them separate; while a few months afterwards it may possibly be received from a neighbour's house, merely by an accidental visit for a few minutes. In the one case, there was no predisposition in the habit to receive the complaint; in the other, the altered state of the atmosphere has, perhaps, produced such a predisposition in a very high degree, and prepared the way for the disease to become a

* Cowan's Vital Statistics of Glasgow, p. 34.

† Rayer des Maladies de la Peau, tom. i. p. 63.

‡ Bateman on Cutaneous Diseases, p. 70.

very general epidemic. What this peculiar state of the atmosphere is has not been very accurately ascertained."* Now, although it be granted that cleanliness and ventilation have somewhat less effect in preventing the spread of smallpox, measles, and scarlet fever, than in checking the progress of typhus; it has been shown by the above quotations that these diseases differ from one another in points equally material. It follows, therefore, that scarlet fever is regulated by a law similar to that of typhus, in being little contagious under certain circumstances; and that though cleanliness and ventilation may not prove an antidote equally efficacious to the contagion of smallpox, measles, and scarlet fever as to that of typhus; yet to exclude the latter from the class of contagious fevers from this circumstance, would involve also the exclusion of scarlatina for an equally strong reason. In reasoning upon this subject, it does not seem difficult to conceive that one species of effluvium may be harmless, if diluted with a certain proportion of atmospheric air, while another may retain its virulency under similar circumstances; or that one species of effluvium may adhere with tenacity to every kind of clothing, while another is absorbed most readily by filthy garments, or by the deposits which are formed on the skin of an uncleanly person. We are in possession of no experiments which tend to prove such an opinion; but there is one analogy which will occur to every medical practitioner in vaccination. It is well known that if too much blood be drawn during the process of vaccination, the effect is very frequently prevented; and this is always explained on the principle, that the vaccine virus is diluted too much with the blood, as the same result follows when it is mixed with water. We have many analogies among the gases, such as carbonic acid, carburetted hydrogen, &c., to prove not only that when diluted to a certain extent with atmospheric air they may be respired with safety, but that each gas has its own peculiar law respecting the requisite proportion of dilution that is required for that purpose.

The majority of French physicians are of opinion that typhoid fever is not contagious, and this belief was almost universal until M. Bretonneau published a contrary opinion. The following quotation from M. Chomel will perhaps account, to a certain extent, for the opinions of the French physicians on this subject: "Another circumstance contributes with us to render the transmission of a contagious malady difficult, particularly the typhoid disease, that in our hospitals everything connected with cleanliness and ventilation is in the most perfect condition, and that the typhoid patients are never united, either in the same establishment or in the same ward, while their number is always very small, when compared with the number of those affected with other diseases; so that none of the conditions are present which favour contagion. It is the same with smallpox, and no one disputes its contagious character. In the wards of our hospitals there are persons frequently affected with smallpox, and there are often individuals who have not been vaccinated, or who not having undergone variola are susceptible of contracting the disease; yet few instances of its transmission are evident. It is also very rare that the transmission of measles or scarlet fever from one subject to another, in

* Good's Study of Medicine by Cooper, vol. iii. p. 19.

the *Hôpital des Enfans de Paris*, can be verified, which even presents in some circumstances the most favorable conditions for the transmission of these diseases."* At page 98 some tables are given which tend to show the connexion of filthy habits with typhus contagion.

2. *Typhus generally attacks individuals only once during their lives.* The second law of contagious fevers is that they only affect persons once during their lives.

We believe this law to be completely established, and though there are instances of smallpox, measles, and scarlet fever affecting individuals more than once in their lives, yet these may fairly be considered only as exceptions to the general rule.

Before bringing forward the facts upon which the claims of typhus to be comprehended under this law may be founded, it is necessary to state that the evidence is by no means so clear and satisfactory as it is in smallpox, measles, and scarlet fever. These three last-mentioned diseases cannot, in the present day, be readily confounded with any other; for their diagnostic marks are very precise and definite; while the several kinds of continued fever have hitherto not been accurately ascertained, and have sometimes been considered merely as varieties of typhus; hence the difficulty of establishing the application of this law to any one of them. We hope, however, it will appear from the quotations which shall presently be adduced, that the approximation of typhus to this law is so near as to preclude, in all fairness, its exclusion. M. Chomel, after remarking upon the number of persons that are seized more than once with pneumonia, states that "In the typhoid fever, on the contrary, notwithstanding the care with which the patients had always been interrogated on this point, no one, among 130 persons who had been received at the *Clinique* affected with the disease, gave such a statement as could lead to the presumption that he had ever before laboured under it; on the contrary, most of them asserted that it was the first time they had been ill."† He elsewhere adds: "We have already stated that the typhoid fever, in ordinary circumstances, affects the same individual only once. This is the result of all the facts hitherto collected. Since we began to make special researches and conclusions respecting this disease, no authentic example to the contrary has been observed, although the number of cases which are observed be very considerable, and examples of its return ought to be met with, if the malady were susceptible of being reproduced many times in the same person."

In interrogating our patients, we have always taken care to turn their attention from this quarter, but they have never answered in such a manner as to induce us to believe that they had laboured under the same disease; and after all, though some contrary facts should be met with in so frequent a malady, these exceptions which are little numerous are nothing extraordinary, and do not overthrow the species of law which has been announced. Smallpox, scarlet fever, measles, which most generally attack the same individual only once, sometimes return, especially during epidemics of these diseases; it will not be more astonishing if

* Chomel, Leçons de Clinique Médicale, tom. i. p. 321.

† Ibid., p. 309.

some examples of a return of the typhoid affection be met with. This circumstance is, then, a very important fact, for there are only a small number of diseases that attack the same individual only once, and amongst these maladies, there is none which is not evidently contagious; the typhoid fever will, then, be the only exception to this kind of law if it be not contagious like the diseases with which it has this important point of agreement. In the mean time we ought to observe that, though all the diseases which attack the same person only once are contagious, it does not follow that all those that are transmitted from one individual to another attack only once, many among them, as syphilis and the itch, are reproduced indefinitely."*

Dr. Lombard, of Geneva, when describing the difference between the continental and British typhus, states that "in one remarkable point, however, I believe they agree, I mean the fact that no one is known, or at least is very rarely known, to have the eruptive typhus twice. With us such instances are scarcely if ever met with, and I am informed that with you a person once attacked with typhus, attended with the measles like eruption, may safely calculate upon immunity from the disease for the future."†

Dr. Perry, of Glasgow, states as one of his conclusions respecting typhus fever, that "contagious typhus is an exanthematous disease, and, like smallpox, measles, and scarlet fever, during its course produces some change on the system, by which the individual having once undergone the disease is (as a general rule) secured against a second attack, and may with impunity expose himself to the contagion of typhus, if he continues to reside in the same country in which he previously had the disease. In those cases which are exceptions to the general rule, the disease appears in a mild and modified form, the crisis taking place on the seventh, ninth, or eleventh day." The same author states that this conclusion as well as the others in his paper are "the result of careful observation in upwards of 4000 cases."‡ Drs. Barker and Cheyne, who had the most extensive opportunities of ascertaining the history of typhus, seem to entertain opinions similar to those already quoted. They state that "at the hospital in Cork street, only one physician and the apothecary had an attack of fever; but then most of the physicians of the establishment had laboured under that disease on some former occasion previous to the appearance of the epidemic."§ Dr. Cowan, as already quoted, states that all the gentlemen who have acted as clerks in the Fever Hospital for many years past have been attacked with fever unless they had it previously to their election.

Hildenbrand's opinion on this subject is of a more modified kind. He states that "the miasma of typhus, after having produced the fever, destroys almost always for a certain time the susceptibility to a similar contagion; nevertheless, it destroys it rarely for the whole of life, as does smallpox, measles, &c. It has, however, under this resemblance some analogy with the virus of these diseases, whilst on the contrary it

* Chomel, Leçons de Clinique Médicale, tom. i. p. 333.
† Dublin Journal of Medical Science, vol. x. p. 22.
‡ Edinb. Med. and Surgical Journal, vol. xlv. p. 67.
§ Barker and Cheyne on Fever, vol. i. p. 135.

totally differs from the syphilitic virus, which when once introduced into the human body, always favours more and more a similar contagion."*

The following table shows the answers to questions which were carefully put to patients who were admitted into the Glasgow Fever Hospital from November 1st, 1838, to November 1st, 1839. It includes the whole of the patients affected with eruptive typhus, from whom answers were obtained relative to any former affection with fever, as evidence from decided cases only could be made available in the elucidation of this point:

| | Males. | Females. | Total. |
|---|---|---|---|
| Not previously affected ... | 284 | 251 | 535 |
| Previously affected ......... | 33 | 41 | 74 |
| | | | 609 |

This table shows that out of 609 eruptive or decided cases of typhus there were only 74 persons who stated that they had previously laboured under fever. This part of the evidence may be reckoned positive; for individuals of all intellectual capacities remember a remarkable circumstance of this kind. On the other hand, the evidence respecting the nature of the former fever or affection is the converse of this; for only in a very few cases can it be correctly ascertained; and when we take into account the various diseases which are confounded with typhus (as shall be afterwards shown), such as bronchitis, pneumonia, pleurisy, intestinal affections, febriculous or short fevers, and the numerous ailments of childhood, this small number can be satisfactorily accounted for.

It appears, therefore, that the evidence which can be produced to bear on this point, although not very extensive, decidedly supports the opinion that eruptive typhus fever affects individuals as a general rule only once in their lives; and it is to a considerable extent corroborative of this opinion, that almost all the clerks and nurses of the Glasgow Fever Hospital for the last six or seven years have had typhus characterized by the eruption, and not one of them, as far as we have been able to learn, have ever had it since; while almost all of them consider themselves now perfectly secure against a second attack, although constantly exposed to the effluvia arising from fever patients.

3. *Typhus is characterized by an exanthematous eruption.* The third characteristic of the contagious exanthemata is an eruption which has a regular rise, progress, and decline. The exanthematous eruption or rash which is peculiar to typhus fever has only been accurately attended to within these few years as a diagnostic symptom. It was, however, noticed by Rogers, in the fever which prevailed in Ireland during the year 1731, and one of the characteristic symptoms is described as "a universal efflorescence of petechiæ;"† also by Huxham in 1734-5, Sir John Pringle in 1750, &c.

No particular conclusion can be drawn from these authors' account of it; but when taken along with their general description of the disease,

* Hildenbrand, de Typhus contagieux, par J. C. Gasc, p. 118.
† Barker and Cheyne on Fever, vol. i. p. 4.

the opinion is corroborated that it was the very same affection that is so characterized at the present day. Hildenbrand described it in 1806 more particularly than any previous author. He states that it makes its appearance about the fourth day of the disease on the breast, loins, back, thighs, and arms, as being more warm, but sometimes on the face; that it is so much more abundant as the eyes are red. He also remarks that petechiæ may exist with or without the eruption, are not indispensable phenomena, and are only developed in certain conditions. He farther observes that the *exantheme* is sometimes not present in those cases of typhus which are irregular in their progress.* The typhoid eruption was also a very general characteristic of the epidemic fever which prevailed in Ireland during the years 1817-18-19. Dr. Bracken makes the following statement: "Of about 250 cases which fell under my care in November and December of that year, the majority had eruptions of spots of various appearance as to size, shape, and colour. They were generally of a diffused appearance, gradually shading off, and insensibly disappearing, and of the size of a grain of hemp-seed, but sometimes much larger or much smaller. The distinct, well-defined petechiæ were frequently seen of a bright brown or purple colour. The shoulders seemed to be more frequently affected by these eruptions, but the whole surface of the body was often covered by them."† Drs. Barker and Cheyne give the following account of the eruption, as deduced from reports received from several parts of Munster: "As the disease advanced it was observed in most or all parts of the province that eruptions of different kinds, either closely allied to or varieties of those termed petechial, very generally accompanied it. In some instances the eruption was papular, or a motley appearance of the skin, or a rash somewhat resembling measles showed itself. At Cork, Dr. M. Barry remarked that in the species of fever which he terms synochus, petechiæ seldom occurred earlier than the fourth or fifth day; but his observation, if it does not express it directly, at least implies that their occurrence was frequent. They were generally of a bright red colour, sometimes small, at other times large. He did not consider them dangerous, nor find it necessary to abstain from those measures of depletion which were useful when high excitement prevailed. In a communication from Clonmell, Dr. Fitzgerald states that petechiæ occurred in four cases out of five. At Listowel, petechiæ was so common that Dr. O'Connell did not see six cases of fever unattended by a petechial eruption, which often appeared early in the disease." In the account of Connaught, the same authors state that "an early eruption of petechiæ, which were often to be observed on the third or fourth day or even earlier, and were visible for four or five days, was a general symptom of the disease; when petechiæ appeared thus early, they were not indicative of any malignancy." In the report for Ulster, it is stated that petechial eruptions were very common and that they occurred early. For Leinster, the same reporters state that one physician observed the petechiæ in seven cases out of ten, some thought them more general than they had been on any former occasion, and others represented them as universal. They appeared on the

* Hildenbrand, de Typhus contagieux, par J. C. Gasc, p. 53-4.
† Barker and Cheyne on Fever, vol. ii. p. 231.

third, fourth, or fifth days, continued visible for four or five days, and were often remarked in the mildest cases.*

The typhoid eruption, however, excited very little attention among the authors who wrote upon the epidemic that prevailed in Britain about the same period as in Ireland; and even up to a much later period it is only noticed in a cursory manner in our treatises on fever, and not as a diagnostic mark of great value. Dr. Alison in 1827 described it as a very frequent symptom of the epidemic which prevailed in Edinburgh about that period, occurring in a majority of the cases, and remarked that these eruptive fevers formed the connecting link between continued fever and the contagious exanthemata.†

M. Louis, who published his admirable work on gastro-enteritis or typhoid fever in 1829, states that "he has observed this eruption in twenty-six out of thirty-five cases, where it has been searched after, without saying that it was not present in some others; many of the persons in whom it was present had come to the hospital after the twenty-fourth day of the disease, at a period when the spots had perhaps disappeared."‡ M. Chomel gives the following excellent description of the typhoid eruption: "Usually from the seventh to the ninth day the eruption peculiar to typhoid fever makes its appearance, which consists in minute rose-red spots, disappearing on pressure from half a line to two lines in diameter, of a circular form, without elevation or scarcely raised above the level of the skin, dispersed over the abdomen, sometimes on the chest, less frequently ou the thighs, the arms, and forearms. These little spots are so much the more distinct as the skin is white; in persons who have brown skins they are sometimes distinguished with difficulty. Their number cannot be determined because they are not all equally apparent; but in order to furnish a characteristic of the typhoid affection they ought at least to be from fifteen to twenty. When there are only two or three, no value can be attached to their presence. The eruption does not make its appearance on all points at once; often, after having noticed for three or four days some rose-coloured spots upon the abdomen, but in too small number to be considered as important, they are found all at once very numerous upon the chest and belly, sometimes upon the thighs, the arms, the back, and even the face, though very rarely. Its duration is not always the same; in some cases, after two or three days, there is no vestige of it; at other times it persists during twelve or fifteen; but in the latter case it consists of many successive eruptions; for each rose-coloured spot is usually visible for three or four days only and sometimes less; and at the end of this time it disappears altogether, after having attained a colour less vivid. These spots present, at most, a slight elevation on the surface of the skin, but they never have a conical form or vesicles at their apex. They rarely appear before the eighth day after the invasion of the disease. The following are the results of observations collected in our wards during the years 1830-1-2. Among seventy cases of typhoid fever, where the presence or absence of rose-coloured lenticular spots was carefully established, in sixteen cases, at no period of the

* Barker and Cheyne on Fever, vol. i. pp. 426, 454, 465, and 483.

† Edinb. Med. and Surgical Journal, vol. xxviii.

‡ Louis, de Gastro-Enterite, tom. ii. p. 231.

disease could traces of this eruption be found; from which it may be inferred that in about one fourth of the persons seized with the typhoid affection this eruption is wanting."*

Chomel found that among fifty-four cases none presented the eruption before the sixth day, and in two it appeared after the thirty-sixth. This, he states, is confirmed by the observations of M. Louis, which were made on a much larger number of patients. He attaches great value to the eruption, as a diagnostic mark of typhoid fever, as it is as rare in other acute diseases as it is common in this.

Dr. Roupell states that in St. Bartholomew's Hospital, London, the eruption in typhus occurs in seventy out of every 100 cases.† Dr. West, in his account of the typhus exanthematicus as observed in St. Bartholomew's Hospital, states that "forty-two cases presented the peculiar measle-like eruption described by so many authors, which in all those cases in which I have been able accurately to note the date of its appearance, first showed itself from the sixth to the eighth day, generally on the former. It appeared in one instance on the fourth and another time on the fifth day; but I never saw it make its first appearance after the eighth day, though it was still visible on several patients admitted on the fourteenth, and on three who came to the hospital on the twenty-first day of the affection. Of the eighteen cases in which no eruption was observed, five only were admitted before the eighth day of the disease; it is, therefore, very probable that the eruption had existed in some of these patients but had disappeared before their admission."‡ Dr. Cowan has investigated the frequency of the eruption in the Glasgow Fever Hospital on upwards of 2000 cases, during the year 1835-6; and his results are the following: "At the close of the year, in 76·16 per cent. of the males, and 71·77 of the females, the typhoid eruption had occurred, giving as an average of the whole cases 73·99 out of every 100 admitted."§

Dr. Craigie found the typhoid eruption only in seventy-nine among 169 cases in the Edinburgh Royal Infirmary;|| while Dr. Henderson discovered it in 108 cases out of 130 in the same insitution at a subsequent period.¶

In the Glasgow Fever Hospital, from May 1st to Nov. 1st, 1839, during which time the presence or absence of eruption was carefully noted, the proportion was as follows:

| | Males. | Females. | Total. |
|---|---|---|---|
| Cases with Eruption ................. | 224 | 217 | 441 |
| Cases without Eruption or doubtful | 130 | 120 | 250 |
| | | | 691 |

This table includes every case, with the exception of smallpox, measles, hooping-cough, and scarlet fever.

* Chomel, Leçons de Clinique Médicale, tom. i. p. 18.
† Roupell on Typhus, p. 35, 1838.
‡ Edinburgh Medical and Surgical Journal, July, 1838, p. 140.
§ Cowan's Vital Statistics of Glasgow, p. 26.
|| Edinburgh Medical and Surgical Journal, vol. xxvii. p. 301.
¶ Ibid. October, 1839, p. 437.

Dr. Peebles, in 1835, gave a very minute and excellent account of exanthematous typhus, and states, as the result of a minute enquiry into the subject in Great Britain and on the continent, that "he has found the eruption as constant as any exanthema of other eruptive diseases."*

It appears, therefore, that the eruption is present generally in from 70 to 75 out of every 100 patients that are admitted into fever hospitals in this country as well as in France. It must be well known, however, to every hospital physician, that cases are frequently admitted as continued fever, that are found, on examination, to be other diseases, and which are usually included in the total enumeration; but this point shall be further illustrated in another part of the essay.

It is also well known that many cases of fever are admitted at a very late stage of the disease, as may be proved by statistical tables and also from the great number of deaths that occur on the first, second, and third days after admission; hence it is extremely probable that the eruption has disappeared in a certain proportion of those who have the other decided symptoms of typhus. There is one fact, however, which powerfully supports the opinion that contagious typhus, in the great majority of cases, particularly in adults, is attended with the eruption, namely, that almost all the instances of fever which have occurred during the last six or seven years among the physicians, clerks, nurses, &c. of the Glasgow Fever Hospital, have been accompanied with this exanthema. We have made careful enquiries respecting this point, and have only heard of one or two exceptions amongst at least 100 cases. We do not, however, mean to maintain that typhus fever cannot exist without the presence of this eruption; on the contrary, we have repeatedly attended families where the majority only of those affected were so characterized, and we have remarked this two or three times, when several of a family were sent into the hospital.

The want of regularity in the appearance of the eruption, and its persistency in regard to time, has been considered opposed in analogy to that of smallpox, measles, and scarlet fever; and certainly, though these three diseases are more regular in their characteristic eruption than typhus, yet in scarlatina anginosa the eruption is frequently irregular or altogether absent. Dr. Tweedie, who must have treated scarlet fever extensively in the London Fever Hospital, makes the following statement: "Indeed, we are inclined, from our own experience, to affirm that the scarlatina simplex, scarlatina anginosa, and the scarlatina or angina maligna, and the sore throat without efflorescence on the skin, are merely varieties of one and the same disease."† He also quotes the results of Dr. Willan's experience during an epidemic scarlatina in the year 1786. "Of 251 cases, there were 152 of scarlatina anginosa, 42 of sore throats without eruption on the skin, and 39 of scarlatina maligna."‡ Rayer states that "often it does not appear until the third day, and is not dispersed so constantly upon the whole surface of the body. ...... It is sometimes entirely effaced on the day of its appearance, and is developed anew at a period more or less near.........The appearance of

* Edinburgh Medical and Surgical Journal, vol. xliv. p. 373.

† Cyclopædia of Practical Medicine, vol. iii. p. 647. ‡ Ibid., p. 653.

the exantheme is tardy; its tint is feeble and livid; it is interspersed with petechiæ, and its duration is uncertain. It appears and disappears many times."* It appears, therefore, that scarlet fever often differs from smallpox very materially in the regularity of its eruption; for that of the latter disease is extremely regular and almost unvarying in its rise progress, and decline; while in the former it is frequently absent, and in other cases so evanescent as not to be distinctly recognized. The eruption characteristic of typhus again differs from that of scarlet fever, in being still less regular than it; but there is not a greater difference, if there be not less, in this respect, between scarlet fever and typhus than there is between scarlet fever and smallpox.

4. *Typhus cannot be checked in limine.* It has also been stated by authors, and it is a prevalent opinion among medical practitioners, that typhus can be checked in its early stages, and that in this respect its law is totally different from the exanthematous fevers. If this opinion were correct, the analogy between these affections and typhus would be greatly diminished, though not completely undermined; for it is not contrary to experience to suppose that some agent may be discovered that might be capable of modifying or destroying the poisonous principle that is lodged in the body. Those, however, who believe in the possibility of checking typhus *in limine*, have assumed a false premise, at least one which is not admitted, from which they draw their conclusions, namely, that every febrile affection which resembles this disease in its early symptoms is identical with it. Now it is well known to every medical practitioner that many of those febrile attacks which arise from disturbance of the digestive functions and from vicissitudes of temperature are attended with the same symptoms as typhus in its early stages; and yet they will subside in a few days under every variety of treatment, and frequently without any treatment at all, at least such as could produce any effect on the system. If typhus fever, which is frequently so prevalent, could be checked in its progress by the means which are generally employed for that purpose, namely, bleeding, purging, sweating, &c., this doctrine would long ere now have been established with the same certainty as that peritonitis or pneumonia can be checked by a similar system of treatment; and yet the disease proceeds onwards in its course, unrestrained by the heroic, but occasionally injudicious attempts to arrest it. If, then, there be febrile affections which subside in a few days under every variety of treatment, and often without any possessing a curative operation, it follows that those who make the assertion that they can check typhus *in limine*, should prove that their cases did not belong to the febriculæ we have referred to; or what would amount to the same thing, make their experiments upon an unequivocal example of the disease, namely, one characterized by the eruption, and demonstrate that they can stop its career.

*Crisis of typhus is pretty regular in cases not complicated.* A second objection has been brought forward against the inclusion of typhus among exanthematous fevers, namely, that it has no regular crisis like these last-mentioned diseases. This objection does not seem to have much weight

* Rayer, Traité de Maladies de la Peau, tom. i. pp. 59-60.

attached to it; and to give it any degree of importance, it would be necessary to prove that all the other fevers of this order are uniformly characterized by a crisis on a particular day. Now, what are the facts connected with the history of this point in scarlet fever. Dr. Bateman states that the rash in scarlatina anginosa does not always appear on the second day, as in scarlatina simplex, but not unfrequently on the third; nor does it so constantly extend over the whole surface, but comes out in scattered patches, which seldom fail to appear about the elbows. Sometimes, too, it vanishes the day after its appearance, and reappears partially at uncertain times, but without any corresponding changes in the general disorder; the whole duration of the complaint is thus lengthened and the desquamation is less regular...........The same author, after describing the dangers which result from hemorrhage, diarrhœa, &c., in malignant scarlatina, states that "even those who escape through these dangers have often to struggle against many distressing symptoms for a considerable length of time, such as ulcerations spreading from the throat to the contiguous parts, suppuration of the glands, tedious cough and dyspnœa, excoriations about the nates, &c., with hectic fever."* When treating of measles, Rayer remarks that "it is never the exantheme which compromises life. The gravity of the evil depends upon the internal inflammation which accompany or succeed it. —The appearance of the measles before the third day, the sudden disappearance or the leaden redness of the spots, the appearance of petechiæ, much difficulty of breathing are severe symptoms. They are often characteristics of bronchitis and pneumonia, the existence of which is easily ascertained by auscultation and percussion of the chest. ...... When the symptoms of gastro-pulmonary inflammations, which accompany the exantheme of measles, are little intense, and when it travels over its periods *easily and regularly*, the treatment of the disease is very simple."† It is obvious from these quotations, and it is well known to every experienced practitioner, that the crisis of measles and scarlet fever varies considerably in different individuals, being pretty regular and early in the simple cases, and more or less protracted and irregular in those that are complicated with any organic affection. In typhus fever, uncomplicated with any serious organic disease of the head, chest, or abdomen, the crisis occurs very frequently about the same period in persons of a similar age; for young persons, as a general rule, pass through the disease more quickly than those more advanced in life.

Chomel states that the crisis or amelioration of the symptoms in sixty-eight cases, occurred in fifty, or in about three out of four, from the fifteenth to the thirtieth day.‡ Dr. Arthur Thomson states that the average duration of 2630 cases was twenty-seven days; and this calculation was made from cases described and enumerated in the works and papers of Drs. Bateman, Welsh, S. Smith, Latham, and Craigie.§ There is certainly considerable irregularity as to the period when the crisis takes

* Bateman on Cutaneous Diseases, pp. 73 and 85.
† Rayer, Traité des Maladies de la Peau, p. 24.
‡ Chomel, Leçons de Clinique Médicale, vol. i. p. 44.
§ Edinburgh Medical and Surgical Journal, July, 1838, p. 109.

place in typhus in different individuals; at the same time, it may be remarked that a majority of patients begin to ameliorate within a certain period, and the reports from the various authors already quoted in regard to this point do not differ very materially as to the mean duration of fever, showing, even with imperfect statistics, a near approximation to some law by which it is regulated. It ought to be observed, however, that the evidence obtained from public hospitals is still very uncertain; for unless very careful and repeated enquiries be made to the patient, no satisfactory or accurate answer can be obtained, as to the period when the disease commenced; for he is often partially incoherent, and in almost all cases, more or less confused in his ideas; and even though there be an opportunity of questioning his friends, more or less of cross-examination is generally required to elicit a correct answer.

A degree of uncertainty also arises from not calculating the crisis always at the same period. Some, as Chomel, Mills, Stoker, &c., calculating the termination of the disease from the commencement of the convalescent stage; while others have included the whole period of the patient's residence in the hospital in its duration. It is obvious that great discrepancy must arise from such a different method of calculation; for a patient is often a week and sometimes two weeks in an hospital after the period of convalescence commences. Another uncertainty on this point has arisen from not classifying patients according to their different ages, such as is employed in calculating the mortality of typhus at the different periods of life; for if the disease be shorter in its duration, as it certainly is, in young persons than in those more advanced in life, it is impossible to expect uniformity by arranging the whole together. It would also contribute to elucidate this point, were the duration of the undoubted cases, namely, those characterized by the eruption, classified separately; as by this means, the duration of typhus would not be confounded with that of other continued fevers, and this method might also be made available as one of the means of diagnosis.

The very frequent complications of typhus with organic affections in the different cavities of the body is another reason amply sufficient to account for a considerable portion of its irregularity as to termination; and as these complications occur more frequently in this disease than in smallpox, measles, and scarlet fever, it follows that allowance should be made for its greater irregularity on these accounts; as it has already been shown by quotations from authors that some of the exanthematous fevers are also rendered irregular and protracted by organic complications.

Dr. Arthur Thomson gives the following table of the complications of fever compiled from cases related by Drs. Smith, Tweedie, Alison, and Craigie, and it shows that the complicated varieties are much more numerous than the simple or uncomplicated.

| | | |
|---|---|---|
| Simple fever | | 374 |
| Fever with cerebral complications | | 375 |
| thoracic | do. | 264 |
| abdominal | do. | 180 |
| mixed | do. | 308 |
| | | 1501 |

5. ***Relapses in typhus do not occur after complete convalescence, unless some local disease be present.*** The occurrence of relapses in fever is also brought forward as an important difference between it and the contagious exanthemata; and almost every author who has written on this subject mentions them as more or less frequent at different periods and during different epidemics. They do not seem to occur frequently in the typhoid fever, for Chomel does not make any particular reference to this point; and he merely states that the convalescence is sometimes prolonged, and that troublesome results are sometimes the consequence of satisfying hunger.

In considering this subject it is necessary, however, to observe that relapses, according to most authors, do not mean a return of the fever after complete convalescence, but a return of the symptoms with their former intensity, after a partial recovery, or what in the majority of cases might more properly be called a remission of the disease; for it is perfectly obvious that if the febrile symptoms had only been meliorated, but not removed, that the disease was still present in the system.

That relapses do occur, in a small proportion of the continued fevers of this country, after complete convalescence, we have no manner of doubt; but certainly this occurrence is rare in typhus, without the co-existence of some organic lesion sufficient to account for the return of the febrile symptoms. A considerable number of what are generally called relapses, particularly during the prevalence of an epidemic, consist of cases which have been admitted with fevers not of a typhoid kind, such as febricula, bronchitis, and those arising from gastric or intestinal derangement; and have caught the contagion of typhus during their residence in the hospital. That this occurs, as a general rule, in the fever hospital of this country we are not in possession of facts to prove; but that it has very frequently occurred in the Glasgow Fever Hospital there can be no doubt; and during the late epidemic it was considered very dangerous to retain a patient long in the house after his convalescence from a febriculous affection.

The following table shows the number of relapses or secondary affections that occurred after complete convalescence among 686 cases, admitted into the Glasgow Fever Hospital from May 1st to November 1st, 1839, erysipelas being excepted:

| (Males.) Secondary Affections. | | | | | | | | | |
|---|---|---|---|---|---|---|---|---|---|
| | Typhus. | Cynanche Tonsillaris. | Apoplexy. | Peritonitis and Int. Ulcers. | Œdema Glottidis. | Intestinal Fever. | Ulceration of Uvula. | Delirium Tremens. | Total. |
| Typhus ............ | ... | 1 | ... | 2 | ... | ... | 1 | ... | 4 |
| Febricula ...... | 2 | ... | ... | ... | ... | ... | ... | 1 | 3 |
| Intestinal Fever ... | 1 | ... | ... | .. | ... | 2 | ... | .. | 3 |
| Pneumonia ...... | ... | ... | 1 | ... | 1 | ... | ... | ... | 2 |
| | | | | | | | | | 12 |

(FEMALES.) SECONDARY AFFECTIONS.

| | Laryngitis. | Pneumonia & Int. Ulcers. | Synovitis. | Intestinal Fever. | Smallpox. | Roseola. | Pneumonia. | Total. |
|---|---|---|---|---|---|---|---|---|
| Typhus | 1 | 1 | 2 | ... | 1 | ... | ... | 5 |
| Febricula | | | | | | | | |
| Intestinal Fever | ... | ... | ... | 1 | ... | ... | ... | 1 |
| Pneumonia | | | | | | | | |
| Roseola | .. | ... | ... | ... | ... | 1 | ... | 1 |
| Bronchitis | ... | ... | ... | ... | ... | ... | 1 | 1 |
| | | | | | | | | 8 |

Total of Males and Females = 20.

It appears from these tables that among the cases of typhus there was not a single relapse into the same febrile state, characterized by a new eruption and the other distinctive marks of this disease; but on the contrary that all the secondary affections were well marked local diseases. It is also shown that two cases of febricula and one of intestinal fever were affected with typhus during their residence in the hospital; and it is probable that more of such cases would have been infected had not the precaution been adopted of dismissing them as early as possible.

In concluding our remarks upon relapses, we shall make the following quotation from Drs. Barker and Cheyne's work, in order to show that one of the most powerful facts in favour of the doctrine of relapses may be explained by the theory we have adopted. These authors state that "as the epidemic advanced and particularly in its latter stages, relapses became very common, insomuch that a very large proportion of those who had been attacked suffered a relapse, and with many this happened several times. . . . . It was remarked at Roscrea that the more early the crisis occurred the greater was the probability of relapse. This observation will apply to every part of this province, for as the epidemic fever approached to a close, a fever of short duration, continuing for about five days, extremely mild and rarely proving mortal, became very frequent, and at this time the tendency to relapse was most observable. On the contrary, after fever of long continuance, it rarely happened that relapse took place."* . . . . The same authors in their medical account of fever in Connaught, state that "relapses were so rare at the commencement of the epidemic that Dr. Veitch, Physician to the County Infirmary in Galway, in his letter of the 6th September, 1817, says that he had not observed one case of relapse out of some hundred cases of fever." In describing the disease as it occurred among the upper ranks in Galway, they state that "petechiæ were universal, insomuch that scarcely a case occurred without them."†

The inferences which may be deduced from these quotations are, 1st. That these short or five-day fevers were either not typhus or their convalesence was only a remission of the disease; for we are not aware of any writer on this subject who describes it as terminating so early.

* Barker and Cheyne on Fever, vol. i. p. 438. † Ibid., p. 455.

2d. Very few of those which were protracted, or which continued to the end of the second or third week, relapsed, which is about the average period for the duration of typhus. 3d. That in Galway, where petechiæ or the typhoid eruption were almost universal, showing the disease to be typhus, not a single case of relapse occurred out of some hundred cases.

### Chap. III. Sources of Continued Fevers, not Typhoid.

Pneumonia, pleuritis, peritonitis, bronchitis, and modifications of these affections are not unfrequently confounded with continued fever, being admitted to fever hospitals as such; and thus the numerical amount of non-eruptive cases of typhus is often considerably increased by the inclusion of these diseases in the list; independent altogether of the two other affections which we are about to describe, and which are generally considered continued fevers, although different from typhus in their prominent features and laws. The first and most prevalent of these two affections has been called febricula, on account of its mildness and short duration, when compared with typhus. The second is prominently accompanied with derangement of the digestive organs, either in the form of constipation or diarrhœa. Chomel makes the following observations, when treating of the diagnosis of typhoid fever: "In effect, various diseases may present, during the first three or four days, a great resemblance to the typhoid affection. Among the diverse morbid states which may at this period present analogous phenomena, we shall find the early symptoms of many eruptive diseases, as smallpox, scarlet fever, and measles; also some catarrhal affections of little intensity: protracted ephemeral fever may be taken for the typhoid inflammatory fever, bilious derangement for bilious fever, exhaustion for the commencement of an adynamic fever, and especially a latent phlegmasia either visceral or venous. . . . . One of the most important characters of the typhoid affection is the duration of the febrile state. As often as the febrile phenomena which can be attached to any appreciable lesion are prolonged beyond a certain limit, eight or ten days for example, there will be already serious grounds for presuming an alteration of the glands of Peyer; and when a disease terminates at the end of some days, we can always be assured, whatever doubts may have existed as to its nature, that it was different from the typhoid affection; and thus all the morbid states, the duration of which does not extend to the tenth or twelfth day, are distinguished."*

1. *Sources of Febricula.* This affection generally commences, like typhus and several other febrile affections, with a rigor, attended by headach, frequency of pulse, heat of skin, flushed face, thirst, moist tongue, generally more or less coated with a whitish fur, and red at point and edges, more or less constipation of bowels, and in the great majority of cases uncombined with any determinate local affection. It is difficult to distinguish this fever from typhus for the first four or five days; but after that the diagnosis may in most cases be made with tolerable accuracy.

If the typhoid eruption be present, there can be no doubt whatever of the nature of the disease; for in Britain this peculiar efflorescence occurs in no other febrile affection that could be confounded with typhus; but

* Chomel, Leçons de Clinique Médicale, tom. i. p. 400.

in a certain proportion of cases it is not present in the latter disease. In cases of typhus destitute of the eruption, there are frequently, however, other symptoms present, even by the sixth day, which are rarely if at all observed in febricula; such as suffused eyes, delirium, or partial stupor, a dry and brown tongue, a dark or dusky hue of the skin. The frequency of the pulse is also a very important symptom in the diagnosis; for in febricula it is rarely above 100, and it generally continues full or of moderate strength throughout the whole course of the disease; whereas in many cases of typhus, the pulse becomes weak, soft, small, or very compressible, at an early period of the disease, and in most cases is more or less above 100 about the sixth or seventh day. Sometimes this fever terminates in one or two days, being described by some authors under the name of ephemera; but more generally symptoms of amendment appear about the sixth or seventh day, and complete convalescence is established, in the large majority of cases, from the sixth to the tenth day. Deafness and desquamation of the cuticle, both of which are frequent characteristics of typhus, are generally absent in this affection. Again, complete convalescence from typhus rarely occurs in adults before the fifteenth day, and is in the majority of cases much later. In children, however, the crisis of typhus generally appears earlier than in adults; but the febriculous affections to which they are liable are proportionally short, often only one or two days in duration. The statistical facts connected with the minimum and maximum duration of typhus have not been very conclusively determined; for, as we formerly remarked, one class of authors terminate the disease when the stage of convalescence begins, while another class do not consider it terminated until the patient is discharged from the hospital; and this discrepancy is still further increased by not carefully classifying the different febrile affections that are admitted into fever institutions and their corresponding duration.

M. Chomel, who seems to have been exceedingly careful in drawing his conclusions only from decided cases of typhoid fever, gives the following statistical account of the duration of the disease, from its commencement to the beginning of convalescence:

| | | | | |
|---|---|---|---|---|
| In 1 | patient | on the | 8th | day after attack. |
| 1 | „ | „ | 9th | „ |
| 4 | „ | „ | 12th | „ |
| 3 | „ | between | 12th and 14th | day inclusive. |
| 10 | „ | „ | 15th and 16th | „ |
| 15 | „ | „ | 17th and 20th | „ |
| 14 | „ | „ | 21st and 25th | „ |
| 11 | „ | „ | 26th and 30th | „ |
| 8 | „ | „ | 31st and 40th | „ |
| 1 | „ | | on the 45th. | |

"If, however," he adds, "we throw aside the cases in which improvement has appeared before the fifteenth day, and those in which it has appeared after the thirtieth, which constitute a small number of exceptions, there remains fifty cases out of sixty-eight, that is nearly three fourths, in which this improvement took place, from the fifteenth to the thirtieth day."* It appears from this table that there were only two out of sixty-eight cases that presented symptoms of convalescence at the

* Chomel, Leçons de Clinique Médicale, tom. i. p. 44.

eighth and ninth day, and if we add five or six days for its complete establishment, the disease, even in this fractional proportion of cases, could not be considered as terminated before the thirteenth or fourteenth day.

This method of calculating the duration of fever, adopted by Chomel and many other authors, is greatly inferior in accuracy to that of marking the patient convalescent when he is actually free of the febrile symptoms, namely, when his pulse is natural, his tongue pretty clean, his sleep tolerably sound, and his appetite moderately good, but still weak and consequently unable to leave the hospital for some days at least. It is quite obvious that the positive character of these four symptoms renders them more fixed, more easily ascertained, and not so likely to be misapprehended as their relative improvement during the first stage of convalescence; and therefore that it is preferable in the determination of this question.

It appears necessary, before presenting our table constructed on this principle, to give one which will show the whole diseases that have been admitted within a certain period into the Glasgow Fever Hospital, namely, from May 1st to November 1st, 1839; as some of our deductions depend upon a fair and impartial consideration of these cases, and as the various statistical points referred to in this section were noted with care.

| | | Males. | Females. | Total. |
|---|---|---|---|---|
| Continued Fevers. | Typhus | 270 | 276 | 546 |
| | Febricula | 32 | 31 | 63 |
| | Gastric or Intestinal Fever | 8 | 7 | 15 |
| | Bronchitis | 14 | 8 | 22 |
| | Pneumonia | 15 | 7 | 22 |
| | Smallpox | 16 | 11 | 27 |
| | Measles | 3 | 1 | 4 |
| | Scarlet Fever | | 4 | 4 |
| | Hooping-cough | 1 | 1 | 2 |
| | Hydrocephalus | 1 | | 1 |
| | Erysipelas | | 3 | 3 |
| | Roseola | | 2 | 2 |
| | Erythema | 1 | | 1 |
| | Hepatitis | 1 | | 1 |
| | Apoplexy | 1 | 1 | 2 |
| | Determination of blood to head | 1 | | 1 |
| | Intermittent Fever | 1 | | 1 |
| | Cynanche Tonsillaris | 1 | 1 | 2 |
| | Syphilis | 1 | 1 | 2 |
| | Delirium Tremens | 1 | | 1 |
| | Suppuration of Kidneys | | 1 | 1 |
| | Phthisis | | 2 | 2 |
| | Dysentery | | 2 | 2 |
| | Mania | | 1 | 1 |
| | | 368 | 360 | 728 |

As a considerable number of the cases in the above table were not continued fevers, it may be necessary to explain one or two points respecting the admissions into the Glasgow Fever Hospital. The facilities of admission have of late been very great, in consequence of there being much more accommodation than was required; and every case, where there was the slightest suspicion of fever, seems to have been sent to this institution, not only from the city, but from its vicinity, to the extent of many miles.

It may be supposed that there is a large number classified as bronchitis and pneumonia; but it requires to be stated that in all the cases of the first-mentioned disease, there were no typhoid symptoms present, and that only two or three were arranged under this division, whose convalescence extended beyond the tenth day, while the greater number of the pneumonic patients were bled from the arm, and the blood found decidedly buffy.

The case marked suppuration of the kidneys was one of peculiar interest. The patient had been delivered of a child about a fortnight before her admission, and was at this latter period found quite comatose, but there were none of the peculiar symptoms of typhus present. The inspection, however, cleared up any doubt that existed as to the nature of the affection, for both kidneys contained numerous small abscesses throughout their whole texture, there was pus in both pelves, in the ureters, and bladders, but no urine in the latter organ.

We shall next present a table, showing the maximum of the pulse and the period of complete convalescence in 181 cases of eruptive typhus and in thirty cases of febricula, that were admitted into the Glasgow Fever Hospital from May 1st to November 1st, 1839, and it includes the whole that were admitted within that period, except two or three, whose convalescence and pulse were not noted, and those that were omitted for reasons to be presently stated.

*Table of the Maximum Frequency of the Pulse in 181 Cases of Eruptive Typhus.*

| Males. | | Females. | |
|---|---|---|---|
| Maximum frequency of Pulse. | No. of Cases. | Maximum frequency of Pulse. | No. of Cases. |
| 86 | 5 | 96 | 12 |
| 96 | 20 | 98 | 1 |
| 100 | 8 | 100 | 3 |
| 104 | 4 | 104 | 5 |
| 106 | 3 | 108 | 23 |
| 108 | 15 | 110 | 1 |
| 110 | 1 | 112 | 3 |
| 112 | 4 | 116 | 3 |
| 116 | 4 | 120 | 17 |
| 118 | 1 | 124 | 7 |
| 120 | 18 | 130 | 10 |
| 124 | 5 | 134 | 2 |
| 128 | 1 | 140 | 4 |
| 130 | 1 | | |
| | 90 | | 91=181 |

Average maximum of pulse in Males = 107·5.
" " Females = 114·1.
" " Males and Females = 110·8.

The five cases in which the pulse is marked 86 were admitted on the seventh, ninth, eleventh, fourteenth, and twenty-first days of the disease, so that it is probable that partial convalescence had commenced at the time the pulse was noted.

*Table showing the Day of the Disease on which complete Convalescence was established in* 181 *cases of Eruptive Typhus.*

| Males. | | Females. | |
|---|---|---|---|
| Day of Disease. | No. of Cases. | Day of Disease. | No. of Cases. |
| 12th | 1 | 13th | 2 |
| 13 | 4 | 14 | 7 |
| 14 | 2 | 15 | 11 |
| 15 | 9 | 16 | 3 |
| 16 | 9 | 17 | 9 |
| 17 | 9 | 18 | 10 |
| 18 | 6 | 19 | 6 |
| 19 | 7 | 20 | 10 |
| 20 | 3 | 21 | 3 |
| 21 | 10 | 22 | 5 |
| 22 | 8 | 23 | 2 |
| 23 | 2 | 24 | 3 |
| 24 | 6 | 25 | 1 |
| 25 | 2 | 27 | 4 |
| 26 | 4 | 28 | 1 |
| 27 | 4 | 29 | 3 |
| 28 | 1 | 30 | 2 |
| 29 | 3 | 32 | 1 |
| | | 34 | 4 |
| | | 36 | 1 |
| | | 44 | 1 |
| | | 54 | 2 |
| | 90 | | 91=181 tot. |

Average Convalescence in Males = 19·7 days.*
" " Females = 21·3 days.
" " days in Males and Females = 20·5

Every case below twenty years of age has been excluded, because the maximum of the pulse varies more from childhood to adolescence than during any other similar period of life; and those who died have also been excluded, as the comparison between the pulse and the recovery would not be uniform in the two diseases, and as the average maximum of the pulse of those cases which terminated fatally was greater than that of those who recovered.

We have taken the eruptive cases of typhus only by which to illustrate the law of convalescence and frequency of the pulse; in order to prevent any doubt as to the nature of the fever from which the conclusion is drawn, and because they constitute the large majority of fever patients. But it may be said that though the non-eruptive cases constitute a small or perhaps only an exceptional proportion of the whole number, they may not follow the same law as the majority, but may be milder, and that the severity of the cases is in proportion to the amount of eruption. Dr.

* Dr. Henderson states that he has seen instances of convalescence on the seventh and eighth days, in which the eruption had existed; but it is not mentioned at what stage of convalescence the calculation was made, and what were the ages of the patients. Edin. Med. and Surg. Journal, Oct. 1839, p. 430.

Henderson supports this opinion, which is founded on the examination of about 200 cases in the Edinburgh Infirmary. We can so far support the author of this paper in regard to the general severity of the cases attended with a copious eruption; but certainly there is no uniform proportion between the two, for we have frequently met with mild cases of typhus in which there existed a copious eruption, and occasionally with some which terminated fatally when there were only a small number of spots. Indeed, reasoning by analogy from scarlet fever, to which typhus has most resemblance in the irregularity of its eruption, we should be led to infer that the intensity of the symptoms would not probably correspond uniformly with the copiousness of the eruption; for cases of scarlet fever have often been found very malignant during some epidemics, although not characterized by any exanthematous eruption, or by one which was only extremely indistinct or evanescent.

There seems to be, therefore, no valid reason why the law of typhus respecting complete convalescence and the frequency of the pulse should not be deduced from the eruptive cases, as they constitute, at least, about three fourths of the whole number, and as there is no uniform proportion between the amount of the eruption and the severity of the symptoms.

*Table of the Maximum Frequency of the Pulse in 30 Cases of Febricula.*

| Males. | | Females. | |
|---|---|---|---|
| Maximum frequency of Pulse. | No. of Cases. | Maximum frequency of Pulse. | No. of Cases. |
| 68 | 2 | 72 | 1 |
| 72 | 7 | 74 | 1 |
| 76 | 1 | 76 | 1 |
| 82 | 1 | 84 | 3 |
| 84 | 1 | 88 | 1 |
| 86 | 1 | 90 | 2 |
| 92 | 1 | 92 | 2 |
| | | 96 | 1 |
| | | 100 | 3 |
| | | 104 | 1 |
| | 14 | | 16=30 tot. |

Average maximum of pulse = 82·8 in Males and Females.

*Table, showing the Day of Disease on which complete convalescence was established in 30 Cases of Febricula.*

| Males. | | Females. | |
|---|---|---|---|
| Day of Disease. | No. of Cases. | Day of Disease. | No. of Cases. |
| 4th | 1 | 3d | 1 |
| 7 | 3 | 4 | 1 |
| 8 | 3 | 5 | 1 |
| 9 | 2 | 6 | 1 |
| 10 | 5 | 7 | 2 |
| | | 8 | 3 |
| | | 9 | 3 |
| | | 10 | 4 |
| | 14 | | 16=30tot. |

Average convalescence = 8 days in Males and Females.

These tables show that, in 181 cases of eruptive typhus occurring in adults, the maximum frequency of the pulse was not below 96, except in five cases; that in about three fourths it was 108 and upwards, and that the average maximum of the whole was 110·8. They also show that only one case of typhus was convalescent on the 12th, and six on the 13th day of the disease out of this number; and that the average convalescence of the whole was 20·5 days. Contrast this with febricula, in which out of 30 cases the pulse did not exceed 100, except in one patient, in whom it was 104; and the average maximum of the pulse for the whole was only 82·8. The convalescence in any of these cases of febricula did not exceed the 10th day, and their average convalescence was 8 days. Are there, then, reasons for maintaining the opinion that these short and mild fevers are specifically different from typhus, in opposition to that of Bateman and many eminent authors? We think there are; for if diseases are to be discriminated by a difference of laws and phenomena, there is certainly in these two affections a wide distinction in their symptoms, and also a distinct line of separation between them as regards the period of their duration and the frequency of the pulse.

This view is supported by the fact that febriculous patients have been frequently affected with typhus during their convalescence in the Glasgow Fever Hospital, which cannot be satisfactorily explained on any other principle than that these two affections are different in their nature. The causes which are generally assigned for febricula also tend to support its disjunction from typhus; for although they have not been sufficiently investigated, yet there is an approximation to something like a proof, that exposure to cold is more frequently an antecedent to this affection than it is to typhus. The following table shows the causes that were assigned for the following cases of febricula:

| Cold. | Uncertain. | Contagion. | | Total. |
|---|---|---|---|---|
| 22 | 28 | 10 | = | 60 |

This result tends to support the popular belief and that of many medical practitioners, that there is a short fever which has sometimes been called "a cold fever," although not necessarily attended by a cough or other pectoral complaint.

It is not probable that this affection is contagious, for though more than one in a family sometimes become affected, this is not generally the case, as in typhus among the lower classes; and it is rare that more than one person from the same house has been admitted for this disease into the Fever Hospital. Besides, the fact formerly stated respecting the almost uniformly typhoid and exanthematous character of the disease in the Glasgow Fever Hospital, when nurses and hospital attendants became affected, has a tendency to support this belief; for cases of febricula are always found associated with typhus in every institution of this kind, when there is no particular restriction respecting admission; and if it were contagious, it is probable that some of the attendants would have been affected with it. And though these short and mild fevers are not generally described and classified separately as to their phenomena and laws, there is abundant evidence existing in the writings of our British and Irish authors to prove that they constitute a greater or less proportion of the fever cases of Great Britain and Ireland. It does not appear to be confined, like typhus when not epidemic, to particular localities, such as large towns, &c., and in all probability it is the most common sporadic fever met with in many country districts. It seems also to be capable of attacking the same individual more than once during his life; and we have in a number of instances attended the same individual within a few years under two different attacks, both having the same characteristics of mildness and shortness. If this view be adopted, it may account to a certain extent for the statements that typhus fever has often been known to affect a person more than once during his life; the one fever being confounded with the other.

We have no facts sufficiently conclusive to bring forward respecting its mortality; but undoubtedly it is very small, unless complicated with a particular local affection: and when a disease which originally has all the characters of febricula becomes protracted, the diagnosis becomes so obscure that any deductions drawn from it are very questionable.

If the analysis of the cases admitted into the Glasgow Fever Hospital during the six months already specified be granted, it will tend to reduce the number of those without eruption very considerably. It is stated at page 21 that there were 250 cases without eruption, and 441 in whom this exantheme was observed. Now among these 250 cases there were 145 other affections than typhus, which, being deducted from 250, leave as those really non-eruptive 105, being above 80 per cent. of eruptive cases. But the number of those cases without eruption might be still farther reduced; for a portion of them were admitted after the tenth day of the disease, when it is presumable that the exantheme might have disappeared, and some of them were verging on convalescence; so that even during the non-epidemic prevalence of typhus, when other febrile affections bear to it a larger proportion than when it is extensively diffused, the number without eruption is not very great; and this fact may account for the opinion which is held by some authors and by many medical practitioners that the exantheme is chiefly characteristic of typhus during the prevalence of an epidemic.

2. *Sources of Gastric or Intestinal Fever.* This febrile affection is very often of an ephemeral kind, lasting only two or three days, and hence it is not frequently met with in hospital practice. Sometimes it results from excesses in eating and drinking which have been repeated in rapid succession; occasionally it is caused by a single indulgence in some aliment difficult of digestion. Persons who have feeble or dyspeptic digestive organs, particularly if the bowels be constipated, are very liable to this affection if their habits be irregular. The person attacked generally feels a kind of malaise for some days previous to the rigor which often ushers in the febrile symptoms; the pulse is sometimes extremely rapid, the skin hot, the tongue is coated with a thick white fur, and there is frequently nausea and an uneasy feeling in the abdomen, which is more or less tumid. The bowels are always either constipated or there is diarrhœa, and when the latter symptom is present, even when the stools are feculent, there is very generally reason to suspect, at least at the commencement of the disease, the existence of solid excrementitious matter in the cells of the colon. This affection is sometimes suddenly terminated by a copious perspiration; but more generally, not until the bowels have been freely unloaded of their feculent contents; and we have repeatedly met with cases of obstinate constipation, in which the febrile symptoms did not completely subside for six or eight days.

In many cases it may be distinguished from typhus at the commencement by ascertaining the antecedent circumstances of the patient, and by the state of his bowels and abdomen. When the diagnosis is doubtful during the progress of the affection, its short duration in the great majority of cases must necessarily distinguish it from typhus. In some instances, however, particularly when diarrhœa is present, the attack is prolonged for a week or two, and sometimes for two or three weeks. In some of these cases there is a tendency to peritonitis, while in others there is reason to suspect some enlargement or ulceration of the glands of the intestines. We are quite aware that such cases, which are not of frequent occurrence, might be called typhus fever without eruption; and in the present state of our diagnostic means this question cannot be solved in a satisfactory manner; but we hope that future investigators will be able to define a line by which they may be distinguished. That intestinal fevers, even those of a protracted nature, are specifically different from typhus may be deduced from the fact that repeated instances have occurred of such patients being affected with eruptive typhus during their convalescence in the fever hospital, and a case of this kind is mentioned in the table of secondary diseases at pages 26-7. Dr. Lombard, of Geneva, in a recent publication, maintains the opinion that there is a bilious fever which is quite distinct from the typhoid fever; but at the same time acknowledges the extreme difficulty of the diagnosis. He states that "the facts collected justify the inference that there are insensible degrees between a simple 'embarras gastrique' and the most severe typhoid fever; but it does not thence follow that there are no true bilious diseases and no true gastric derangements, because we have cited cases of this kind which have terminated by death without presenting any of the lesions characteristic of typhoid fever; only it appears very difficult to distinguish if a mild case of gastric derangement arises from a simple derangement of the alimentary canal, or if, as in a case related of a suicide, it is accompanied by a development of the glands of Peyer. Perhaps in the lenticular eruption may be found the distinctive sign of the intestinal eruption and of the bilious disease. But farther observations are necessary to determine this in at all a satisfactory manner."*

Various other forms of fever than those we have described have been mentioned by authors; but we have seen no reason to believe, either from the account given of them or from our own experience, that there are any other species.

The typhoid eruption has been found in almost the whole of those that were formerly considered distinct fevers; and has identified into the same species, synochus, typhus mitior and gravior, adynamic, ataxic, putrid, spotted, and jail fevers; while synocha or inflammatory fever is admitted to have scarcely an existence in this country, and it is not very easy to conceive how inflammation could exist without the presence of some local inflammatory action.

3. *Bronchitis.* Bronchitis is a frequent complication of typhus fever;

* Clinical Remarks on Bilious and Typhoid Fevers, p. 16.

but this inflammatory affection is also confounded with it and other continued fevers when there is the strongest evidence for believing that the febrile symptoms are solely dependent upon the bronchial inflammation. It may be distinguished from typhus by the affection of the bronchi being almost uniformly the first symptoms of the disease, as indicated by hoarseness, cough, dyspnœa; whereas the bronchitic symptoms in fever are rarely present to any extent at the very commencement. The febrile symptoms in bronchitis are almost always proportionate to the greater or less severity of the bronchitic inflammation, increase as it increases, and decline when it is diminishing, which latter result is often well indicated by the expectoration of yellowish opaque mucus. The duration of bronchitis is also generally shorter than that of typhus, unless it be complicated with some pneumonic inflammation; and when this occurs there may be some difficulty in determining the case. If, however, there be the distinct stethoscopic signs of pneumonia, if the blood be decidedly buffy, and not simply coated with a whitish or greenish-white pellicle, if the skin be of its natural whiteness, if the febrile symptoms be proportionate to the local affection, if there be no stupor, delirium, or suffusion of the eyes; even although the typhoid eruption be not present, there will be a tolerable certainty that the disease is not typhus fever. The effects of a full bleeding are not to be overlooked; for in pure pneumonia, its influence in reducing the frequency of the pulse and the urgency of the other symptoms is generally very decided, which is by no means the result when typhus is associated with this disease.

I. *Alleged sources of continued fevers from putrid effluvia.* It is a well-established fact that the accidental inoculation of the body with decayed or putrid animal matter has produced morbid symptoms, resembling in some respects those of typhus fever, and many medical men have been so affected, after making necroscopic inspections. There is always, however, in such cases extensive local disease of the member inoculated, or a diffused cellular inflammation. According to the researches and experiments of MM. Gaspard, Majendie and Leuret, and Hamont, putrid animal matter, when injected into the veins of healthy animals, proves speedily fatal,* and putrid vegetable matter acts similarly, though to a less degree; while the symptoms induced have some resemblance to those in typhus fever.

The following were the symptoms which were produced in a dog, into the jugular vein of which M. Gaspard injected a putrid solution of fermented cabbage, on the 14th July, 1821. Some hours after the injection of the liquid, there was great malaise, difficult respiration, vomiting, and great weakness. At the end of nine hours a very copious black and liquid stool. On the 15th, the weakness was more considerable; there was lateral decubitus, small and feeble pulse, ardent thirst, natural and abundant urine, free respiration, strong pulsations of the heart, as in aneurism with hypertrophy of that organ. On the 16th, some improvement, less weakness, no pulsations of the heart, great thirst, disinclination to food, fever, and occasionally vomiting of drinks. 17th, the same symptoms. 18th, symptoms aggravated, extreme feebleness, staggering locomotion, excessive thirst, red inflamed eyes and filled with mucus, tumefied nostrils obstructed with mucus, mucous membrane of mouth

* Christison on Poisons, p. 583.

red and phlogosed, a liquid grayish-white stool with some clots of putrid blood, and death at the end of the fifth day of the experiment. On dissection, the lungs were found black and slightly inflamed, but still sufficiently crepitant. The right ventricle of the heart contained an albumino-fibrous concretion, which extended into the superior cava and pulmonary artery. The mucous membrane of the intestines, especially that of the duodenum and rectum, and a portion of the small intestines was violet-red, as if ecchymosed, inflamed chiefly in the form of longitudinal wrinkles and by irregular plates, which variegated the exterior of the intestines before their incision. The mucous glands of the rectum were swollen and very distinct. The mesenteric glands appeared to be engorged with blood and were completely inflamed, the gall-bladder was filled with black, thick, and ropy bile.*

In several particulars the symptoms of a malignant case of typhus were exemplified in this experiment upon the dog; the small quick pulse, the peculiar decubitus indicating great weakness, the black stools, the red colour of the mucous membrane of the mouth and fauces, the injected eyes, and finally the staggering as indicative of delirium. The necroscopic inspection also furnishes some points of resemblance, namely, the inflammatory patches in the mucous membrane of the intestines, the enlarged glands in the rectum, the swollen and engorged mesenteric glands, the black ropy bile; all of which are pathological appearances more or less frequently met with in typhus. M. Majendie found that fatal effects were produced by confining dogs over vessels in which animal matters were undergoing the process of putrefaction; but pigeons, rabbits, and Indian hogs were not in the least injured by a residence in the same cage for nearly a month. He repeated many times this experiment with dogs, and always obtained the same result with one exception; but he states that in this case the dog was acclimated, for the injection of a putrid liquid into his veins had little effect upon him. The symptoms, however, are different from those produced by the injection of a putrid fluid into the veins; for the animals seem to die only from extenuation at the end of about ten days; and the post-mortem appearances are a total absence of fat, of aliments in the stomach, and of chyle in the lacteals; while the mucous membrane of the intestines is inflamed, but less so than when putrid matter is injected into the veins.† It appears, however, well authenticated that workmen employed in peculiar manufactories, and who are constantly exposed to the effluvia arising from animal substances in a state of putrefaction, are not subject to any of those morbid effects which result from the injection of putrid matter into the veins, or, according to M. Majendie, to those which result from exposure to putrid effluvia; there must, therefore, be some other explanation given of the last-mentioned author's experiments, or some unknown concurring circumstances must be required to bring the poison into operation. One of the most remarkable and repulsive manufactories or rather nuisances of this kind is the Chantiers d'Ecarrissage de la Ville de Paris. It is an inclosure of many acres of ground, situated close to the walls of Paris, and has existed for several centuries. Into this receptacle are carried the contents of the necessaries of the city; and the carcases of 40,000 or 50,000 horses, dogs and cats are flayed and cut up there

* Journal de Physiologie, tom. ii. p. 16.

annually. Various parts of these animals are separated and manufactured for sale: the intestines into gut for machinery; the fat is melted for blow-pipe lamps; the flesh, blood, &c. are collected for manure; a compost is made to breed maggots for feeding poultry, and the bones are chiefly used as fuel. Hordes of rats live in this bed of filth and extend their ravages extensively in the neighbourhood. The fetor which arises from it is overpowering, and often spreads to a great distance. It is remarkable, however, and contrary to every preconceived notion that could be formed respecting its salubrity, that the workmen of this establishment and their families are healthy, the most of them being stout and long-lived. This fact has been established satisfactorily by Parent-Duchatelet. This author states that they have all the characteristics of the most blooming health, that in this respect they resemble butchers, and that they seem to attain longevity more frequently than other artisans. Even new workmen employed upon extra occasions, although not acclimated, do not appear to be more susceptible, nor do they become affected with any disease. During the time that cholera prevailed in France, not an écarrisseur was affected with the disease, and not one was sick; and the mortality of the village which is in the vicinity of Montfaucon was very small when compared with that of Paris. He also quotes the innocuous influence of the human bodies which are exhumed to the extent of 200 annually from Père la Chaise, and the exhumations from the cemetery des Innocents, amounting to about 20,000 bodies annually, which occupied three years in the execution, and which was also carried on during the greatest heats of summer.* Dissecting rooms are also situations where putrid effluvia are constantly present; and it has been affirmed that those who are much confined to these places do not enjoy good health, and are subject to fevers. MM. D'Arcet and Parent-Duchatelet state that the most frequent indisposition among those who are engaged in dissections is dyspepsia and diarrhœa, but that this latter affection is frequent among the strangers who arrive at Paris. These authors cite an immense number of authorities of the highest respectability, namely, Boyer, Dupuytren, Lallemand, Roux, Jadelot, Breschet, &c. to prove that dissecting rooms are not insalubrious and are not productive of fevers. M. Andral states that gastro-enterite, meningitis, and typhoid fever are common among the young élèves of medicine during the first year of their residence at Paris; but so little does this depend upon their sojourn in the dissecting amphitheatre, that among those who are affected, there is at least as many seized before they commence their dissections as after this period. He adds that the health of the men employed in handling the débris of dead bodies is similar to that of other individuals.† The workmen employed in the manufacture of strings for musical instruments are exposed constantly to the putrid effluvia of animal substances, arising from their long maceration, and they are not more subject to fevers than other tradesmen.

Butchers, who are believed by some authors to be almost exempt from fevers, are exposed in the slaughter-house to the emanations arising from the putrid blood and other animal fluids, which are frequently allowed to stagnate, and which are sufficiently indicated by the fetid and insup-

* Annales d'Hygiène Publique, tom. viii. p. 139. † Ibid., tom., v. p. 301.

portable odour which issues from these places during hot weather. The atmosphere of whale vessels must be constantly impregnated or rather saturated with the effluvium that issues from large and numerous fishes; yet fevers are not prevalent among the seamen. Majendie states that the most deleterious animal poison is the putrid water of fishes: when some drops of this water are injected into the veins, in less than half an hour symptoms very similar to those existing in typhus and yellow fever are produced, and the animal dies in about twenty-four hours.* It appears from these facts that persons may live constantly amidst the most concentrated putrid animal emanations and yet not contract fever of any type; may enjoy health of the most perfect kind; attain longevity in many instances, and be less subject to some epidemic diseases than the inhabitants in their neighbourhood. It may be asked how are the experiments of M. Majendie and others to be explained upon this view? It does not appear from M. Majendie's experiments that the same symptoms or pathological appearances were produced by exposing dogs to putrid animal emanations, as by injection of a putrid fluid into the veins; indeed, he admits this himself; but adheres to the belief that the effluvium was the cause of death in the dogs subjected to experiment, although no injurious effects were produced on several other animals. Many animal poisons, however, operate differently on different organs and tissues; and this is well exemplified in an experiment mentioned by Dr. Christison, namely, that "a pupil of Professor Mangili swallowed at once the whole poison of four vipers without suffering inconvenience;"† but if a small quantity of this be inserted into a wound, poisonous effects are always produced. From a consideration of the whole evidence that might be adduced respecting this point, it may be drawn as a conclusion that although putrid matters, when injected into the veins of animals, cause death under symptoms similar to those of typhus fever, yet that the effluvia arising from similar matters do not under ordinary circumstances produce any deleterious effects on man. That there are exceptions to this general law we doubt not, such as Olivier being affected with diarrhœa after visiting a cellar filled with old bones, and Chevallier being seized with the same disease after exposure to the emanations from dead bodies; but that the effluvia arising from animal substances in a state of putrefaction constitute any regular source of continued fevers, we think there are no grounds for believing.

II. *Alleged sources of continued fevers from the exhalations of the human body.* Another modification of putrid miasmata has been noticed by almost all authors as a cause of fever, namely, the concentrated exhalations from the human body. Sir John Pringle and other army as well as navy physicians have remarked that fever was often produced in crowded hospitals, especially during hot weather, and also in crowded barracks and in transport ships, when filled beyond a due number. Dr. Tweedie makes the following statement: "The late Mr. John Pearson told me that when he was surgeon of the Lock Hospital, he uniformly observed when more than a certain number of patients were placed in any of the wards, fever became prevalent in the establishment; and that

Journal de Physiologie, tom. iii. p. 83. † Christison on Poisons, 3d Ed., p. 577.

from repeated observation of this fact, he was induced to restrict the number of beds in each ward, and never afterwards witnessed the recurrence of fever in the house."* Dr. Bateman remarks that "if it had not been already demonstrated on the most copious evidence, that the mere accumulation of animal matter in a putrescent state is incapable of generating fever; yet the fact that the closeness of the habitations of the poor, the uncleanliness of their persons, furniture, and apparel, and the accumulating filth in the lanes and alleys which they occupy remain unchanged in all seasons, while epidemic fever appears but rarely and with long intervals of absence, is decisive against the supposition that the latter is engendered from such sources."† It is singular that a writer of such distinguished accuracy, after having drawn so fair a conclusion from the facts connected with the prevalence of fever, should apparently contradict this; for he states in a note that "the morbid and even natural effluvia of the living body, when allowed to accumulate by want of cleanliness and air, are unquestionably common sources of fever, and contribute mainly to its propagations as has been intimated in the preceding note."† The inhabitants of some countries, such as the natives of Kamstchatka, are remarkable for their filth and for living amidst the most foul and putrid effluvia; and yet fever is not known among them. The places they live in are called yourts, which "are sunk seven or eight feet below the surface of the ground, and are covered with a thatched roof in the form of a truncated cone, open at the top; they consist of one small apartment, which usually contains six families, with their utensils and stock of provisions for the winter, the chief part of which is dried fish almost putrefied. . . . . Here they eat, drink, and sleep, crowded promiscuously together, and satisfy all the calls of nature without modesty or restraint, and never complain of the noxious odour that prevails in these habitations."‡

The same mode of living is practised by the inhabitants of the Island of Oonalaska, by the Samoiedes, by the Greenlanders and Esquimaux; and there are no continued fevers among them, although scurvy prevails to a considerable extent. In many parts of Russia the same system of living in filthy and unventilated houses, and in an atmosphere saturated with human effluvia, is practised; yet no febrile disease is the result. Dr. Bancroft quotes the slave-ships as examples, where an atmosphere is more offensively impregnated with human exhalations, excretions, &c. than could probably be found in any other place of confinement, and makes the following statement: "I am fully convinced that fever of any kind rarely occurs on board these vessels, and contagious fever never; though great mortality has frequently happened from other diseases, and more especially from dysentery. . . . . There certainly is nothing in the constitutions of the negroes which exempts them from typhus or contagious fever; on the contrary they have been found as susceptible of it as whites, and considerable numbers of them who were sent from this country and from Nova Scotia to the new colony of Sierra Leone died of it on their passage thither, as will be more fully related in another place."§

* Tweedie's Clinical Illustrations of Fever, p. 83.

† Bateman on Fever, pp. 5, 6, and 7.

‡ Bancroft on Yellow Fever, &c. p. 121. § Ibid. p. 129.

Dr. Bancroft quotes a very remarkable instance of crowding in the Decade frigate during the revolution in France; where 193 persons were crowded to as great a degree as the negroes are in slave-ships, yet not one of them died during a period of ninety-six days.

III. *Alleged sources of jail fever from filth and an impure air.* The breaking out of fever in jails has often been brought forward as a proof of the origin of the disease from filth and an impure atmosphere, being afterwards propagated by contagion; and Sir John Pringle's aphorism is frequently quoted or alluded to by writers on fever, namely, that "the cause seems plainly to arise from a corruption of the air pent up and deprived of its elastic parts by the respiration of a multitude, or more particularly vitiated with the perspirable matter, which, as it is the most volatile part of the humours, is also the most putrescent."

Dr. Bancroft makes the following very pertinent remarks upon this point: "That this fever often exists in them (jails) cannot be denied; but this circumstance can afford no evidence of its having been generated therein any more than the multiplication of vermin in such places could demonstrate the spontaneous generation of these and other insects by the nastiness which favours the deposition and hatching of their eggs.* . . . . Indeed, if it were true that the vegetable or animal matters while decomposing or putrefying could, de novo, generate contagion properly so called, the species or varieties of contagion ought necessarily to have become as numerous and various as the matters so decomposing, and also as various as their relative proportions; every dunghill, every collection of rubbish and filth, ought to be capable of generating the cause of a new disease, and that disease ought to be capable of reproducing itself in other persons."† In estimating the value of the testimony that is generally brought forward to prove the spontaneous origin of fever in jails, and the great improbability of the first person attacked, who may have been resident there for several months, being infected previously to his imprisonment, the remarks which we formerly made respecting the impossibility of tracing the contagion in similar situations, even of diseases universally admitted to arise from that cause alone, will also apply here. And although jails may apparently be the most secure places against the inroads of contagion, from the number of their bars and gates; yet their inmates, from the nature of their offences and their dependent situation, must have a more frequent communication with their friends, either personally or through the medium of clothes, than is generally supposed, and that too frequently with the most filthy and debased of the human race. The question relative to the spontaneous origin of fever in jails seems to be almost solved by the fact that the same cause existing in a variety of other situations produce no disease like continued fever; and it can be proved from the history of other prisons, namely, those in Switzerland, Italy, Russia, &c., and similarly circumstanced as to filth, want of ventilation, &c., that no such diseases were known there. Mr. Howard, who has investigated this subject in his work on prisons states: "If it were asked what is the cause of the jail fever, it would

* Bancroft on Yellow Fever, &c. p. 149.

† Ibid., p. 105.

in general, be readily replied, the want of fresh air and cleanliness; but as I have found in some prisons abroad, cells and dungeons as offensive and dirty as any I have observed in this country, where, however, this distemper was unknown, I am obliged to look out for some additional cause for its production."*

The following fact is worthy of being quoted, as illustrative of the efficacy of cleanliness and disinfection of suspected clothes, &c. in preventing the introduction of fever into jails, which were formerly so much infected by this disease: "In the jail at Cork, the prisoners remained free from fever when it had spread in every direction among the inhabitants of the city. To prevent its introduction, means were employed which deserve record: jail dresses were provided for the prisoners, whose clothes on their admission were removed and heated in a stove, and their persons washed and cleaned; the bedding was occasionally steeped in oxymuriatic acid water and then stoved; patients in whom fever showed itself were immediately removed to an hospital; this system was continued during a year and a half, in the course of which time two prisoners died of dysentery but none of fever; when the medical inspector for Munster made his visit to the jail, the system had for some time been discontinued in consequence of the expense attending the jail dresses, and then fever began to show itself among the prisoners, and a few cases were found in the jail at that time."†

From a review of the whole facts connected with filth and deficient ventilation, it appears that both in the countries where continued fevers prevail and in those where they do not exist, the inhabitants may live constantly amidst this impurity and yet be entirely exempt from any febrile disease of this kind; and that if filth and an impure air were a common source of fever in jails, hospitals, &c., without the influence of contagion, they would produce the same effects in all other countries and localities similarly situated and circumstanced. The opinion, therefore, which is so generally admitted and propagated by many of our first authorities, that fever may arise from common causes, such as putrid miasmata, contaminated air, &c., and yet afterwards be propagated by contagion, receives no support from this presumed source; for though we are not prepared to assert that febrile affections may not, under peculiar circumstances, arise from these causes; yet it is undoubtedly deducible from the evidence that they are not ordinary or even limited though regular sources of the disease in any form.

IV. *Alleged sources of continued fevers from river malaria.* Before concluding this part of the essay, we shall notice an hypothesis which has lately been somewhat confidently brought forward to account for the prevalence of typhus in some large cities, namely, that a peculiar malaria is generated by the animal and vegetable filth which accumulates along the sides of rivers running through large towns, and that the inhabitants who live in their immediate vicinity become thereby subject to fever. We are quite aware that very disagreeable and sometimes fetid effluvia occasionally arise from such situations, particularly during hot

* Bancroft on Yellow Fever, &c. p. 149.
† Barker and Cheyne on Fever, vol. i. p. 97.

weather; but that it is capable of causing continued fever has not even been rendered probable by any satisfactory evidence.

We presume that this point may be determined by the locality of Glasgow; for the Clyde runs through the town, and has a numerous population inhabiting houses close to its banks. This river is also of considerable magnitude; and certainly there is abundance of filth deposited in its bed by the numerous common sewers and public works in Glasgow. We have kept a record of the places of habitation of 934 persons who were admitted into the Glasgow Fever Hospital from January 1st to November 1st, 1839, and have classified the cases in the manner shown in the following table. The town has been divided into seven districts: 1, includes all the streets parallel to the river and close to its banks on both sides; 2, all the streets on both sides of the river, which run at right angles to it and which open into it—these first two divisions are of course excluded from the others; 3, east district of the town, from the cross eastward; 4, west district, from Buchanan street westward, and bounded on the north by Sauchie-hall road; 5, north side, northward of Sauchie-hall road and Rotten row; 6, south side of the river, with the exception of those streets close to its banks or which open into it; 7, centre of the town, from the Cross to Buchanan street, and bounded on the north by Rotten row.

| | Males. | Females. | Total. |
|---|---|---|---|
| Streets close and parallel to river . . | 14 | 10 | 24 |
| Streets at right angles to and opening into river . . . . . . . . | 56 | 51 | 107 |
| East district of town . . . . . . | 140 | 136 | 276 |
| West district of town . . . . | 44 | 54 | 98 |
| North side of town . . . . . . | 44 | 41 | 85 |
| South side of town . . . . | 41 | 22 | 63 |
| Centre of town . . . . . . . | 92 | 106 | 198 |
| From the country, 2 to 14 miles . . | 46 | 37 | 83 |
| | | | 934 |

It is shown by this table that among 934 cases admitted as labouring under continued fever, there were only 24 who inhabited houses close to the river; and when we take into calculation the large population that live upon its banks, this proportion is very small. Again, in those streets which run at right angles to the river, and which open into it, the number is greater; but it must be remembered that most of these streets are long, and that it is only those inhabitants who live at their river termination that are at all exposed to the effluvia. If this be taken into the account, a fractional proportion only of these 107 cases ought to be calculated. The east district of the town, a situation very remote from the river, furnished nearly one third of the whole cases, and it and the centre of the town together more than the half. These facts clearly show that river malaria has no influence in the production of continued fevers in Glasgow, and that it is proportionally as prevalent, if not more so, in other and more central parts of the town.

### CHAPTER II.

### CIRCUMSTANCES FAVORING THE DIFFUSION OF CONTINUED FEVERS.

EPIDEMIC diseases have prevailed from the most remote era of the world, and have, with one exception, hitherto bid comparative defiance to the philosophy of medicine, in its attempts to check their progress or diminish their mortality. Almost every country has its own peculiar pestilence, that sweeps rapidly away its redundant population at periodical seasons; and then its fatal operations ceases, partly from the subjects it can attack being reduced in number, and partly because its laws rendered it progressive from one city or country to another, or because the element that favoured its operation had been changed or modified in its constitution. Many diseases, the contagious as well as the non-contagious, possess the property of becoming epidemic; and smallpox, which is perhaps the most infectious of all febrile affections, is subject to the same law, being rapidly diffusible during some particular seasons, while in others it remains comparatively inactive. The same periodical prevalence of scarlet fever and measles is observed during particular seasons, as of yellow fever, which is generally believed to be a non-contagious disease, and to derive its origin from vegetable malaria generated in a hot climate.

There are four prominent circumstances which favour the diffusion of contagious continued fever:

1st. A humid state of the atmosphere.
2d. Poverty, famine, or food of bad quality.
3d. An accumulation of persons not previously affected.
4th. Filth and deficient ventilation.

We do not mean, however, to assert that these are the only circumstances that operate in the diffusion of contagious fever; for certainly our knowledge of the constitution of the atmosphere, particularly its meteorological and miasmatic qualities, warrants no such conclusion. But if it can be shown that these circumstances generally precede or accompany an epidemic of typhus fever; although they may not account for all the phenomena connected with its extension, yet may so far elucidate the subject as to facilitate the progress of future observations or experiments.

1. *Humidity of the atmosphere, scarcity of provisions, filth, and deficient ventilation tend to diffuse continued fevers.* Almost all authors who have written on epidemic diseases have noticed what is called an epidemic constitution of the atmosphere; but this has in general been so indefinitely stated, far less defined, that no conclusion can be drawn from their descriptions as to the peculiar alterations of which it consists. We shall, therefore, confine our evidence solely to that state of the atmosphere which is either cognizable by our senses or by instruments, as it is impossible in the present state of our knowledge to advance anything but vague hypothesis, upon what authors designate by the term referred to. Typhus fever is a disease peculiar to cold or temperate regions, and it does not appear that it is capable of propagation to any extent in a hot climate; the powerful heat of the sun in such regions appearing to dissipate or destroy its contagious properties. Dr. Bancroft observes that "in voyages to the East Indies, ships remain for a much longer space of time between the tropics, and being also exposed to a higher temperature, the power of heat in destroying typhus fever is in them more decisively manifested, an entire cessation of the disease (however prevalent) commonly taking place before they can reach the Cape of Good Hope. It has indeed never been known, as I am informed, that a single case of this fever had occurred on either side of the Indian peninsula."* The existence of typhus fever, at least its diffusion, seems therefore incompatible with a powerful or tropical heat of the sun, and in that respect it differs very essentially from yellow fever; but the ordinary heat of a temperate climate does not extinguish it or even materially check its progress; for it has often prevailed epidemically during summer as well as winter; though it has been generally observed that the seasons during its prevalence were attended with more than the average quantity of rain. In adducing evidence to prove this point, we shall also include that which establishes the coexistence of scarcity of provisions with its consequences, namely, filth and deficient ventilation, as the descriptions of authors generally comprehend these concomitant circumstances. Drs. Barker and Cheyne state, on the authority of Rogers, that "after the year 1721, there was again an interval of good health in Ireland so complete, that scarcely a case of fever was to be met with; this continued till the year 1728, when, as we learn from Boulter's Letters, there had been three bad harvests in succession. Oatmeal, the chief food of the poor in the north, rose to an extravagant price; in the south, the scarcity was so severely felt, that on the 26th of February there was a great rising of the populace of Cork, who threatened to pull down the Mayor's house. . . . From 1728 fever gained ground, and continued to be epidemical until 1732. . . . In the winter of 1739-40 an intense frost attended with a high wind at S. E. and E. intolerably piercing, set in on the 27th of December, and continued with little interruption till the middle of February." The following season was one of great scarcity, and in "the autumn of 1740, which was unusually frosty, with a continued prevalence of N. and E. winds, fever which had been frequent became epidemical; it did not cease in the winter, and increased most alarmingly in the spring and summer of 1741." O'Connell estimated the mortality of that epidemic at 80,000 persons. "The year 1800 was nearly as unfavorable to the fruits of the earth as 1799. The summer of that year was unusually dry; then followed a short period of uncommon heat; for three weeks or a month the thermometer, when at its greatest height during the day, seldom fell below 70 degrees; cold and wet weather set in about the end of August or beginning of September. Thus a short period of uncommon heat degenerated into an ungenial autumn, yielding in some soils an imperfect produce, whilst in others, the failure of the crops was little less complete than in the preceding season; so that, notwithstanding bounties were granted on the importation of foreign corn, and the distillation of spirits from grain prohibited, yet the price of bread and potatoes, both of bad quality, together with that of every other necessary of life, was raised beyond all precedent. In the autumn and winter of 1800, the inhabitants of this kingdom universally suffered from a contagious fever, in which the troops still continued to participate. . . . . In August, 1801, the garrison of Dublin suffered greatly from petechial fever, which very generally prevailed among all ranks in the metropolis and its vicinity. The epidemic which had now reached its height shortly after began to decline,

* Bancroft on Yellow Fever, &c. p. 510.

but not before the good effects of an unusually abundant harvest, in again furnishing provisions of all kinds to the poor at a moderate rate, had been felt. The winter of 1813-14 had been uncommonly severe, that of 1815-16 did not fall short in severity; but particularly so in the early part of 1816, when the cold was very great in these countries. In the month of February, 1816, the quicksilver in the thermometer in many parts of England fell below 0°. Thus at Northampton, on the 9th of February, 1816, it fell to 4°, and on the tenth to 2·75°. In the neighbourhood of London it fell to 5° below 0°, and during four days of that month it never rose to the freezing point. . . . . From a registry of the weather kept in Dublin, it appears that the mean temperature of the months of spring, summer, and autumn, commencing with February and ending with October of that year, was nearly three degrees and a half below that of the similar preceding period; thus the medium temperature in 1815 was 54·32°, and during the same time in 1816 it was only 50·9°, the difference amounting to 3·42°. In neighbouring countries similar observations were made. According to those of Mr. Howard, in the neighbourhood of London the mean temperature of the same months in 1815 was 53·9°, and in 1816 only 49·9°, the difference amounting to four degrees. . . . . The quantity of rain which fell during the summer and autumn of 1816 was also very great. During the months commencing with July and ending with October in that year, being the season of harvest, the humidity of the atmosphere was almost incessant: rain falling during the greater part of the time in these months. . . . The effects occasioned by unusual cold and humidity and absence of sunshine on the productions of the soil were peculiarly injurious. The harvest of grain was uncommonly late both in this country and in England. Corn remained uncut during the latter parts of October and November, and much of it was altogether lost. The same injurious effects on the quality of the potatoes were produced as upon the grain, and these roots constitute "the principal or only food of the poor" in most parts of Ireland. "The sufferings of the poor at this period did not depend on diminution of vegetable food only; in many or most parts of Ireland, the straw used for bedding was often half decayed, and more than usually disposed to imbibe and retain humidity; perhaps from deficiency of the woody fibres. . . . . Turf or peat is the chief fuel of the poor in this country, and during such wet seasons it could not be cut and dried for use. So great was the scarcity of fuel, that the hedges, which in ordinary times are respected as the boundaries of property, were destroyed, and the trees in many places were denuded of their branches to supply the necessaries of life; a practice at which landed proprietors often connived, sensible that it had arisen from necessity the most urgent. Hence dampness of clothes and bedding, imperfect cooking of food and ventilation of apartments, deficient cleanliness in persons and dwellings, all depending on the want of fuel, contributed to heighten and extend the calamities of the poor of Ireland at this eventful period. The preceding statement refers to the effects of the cold and wet of 1816 chiefly, but the following year was little inferior in severity. The summer and autumn were humid, cold, and ungenial, and agricultural produce, with the exception of potatoes, which were more abundant than in the former year, was almost as scarce as in 1816. . . . . The year 1818 was remarkable for a state of weather the reverse of that in the years immediately preceding. The spring was moist, but the summer set in with unusual warmth, and proved the hottest which has occurred in this country during many years past." To these causes of distress were added a very low price for labour, and extensive failures in trade and manufactures. Drs. Barker and Cheyne also mention the prevalence of fever on the continent of Europe during a series of previous years, and remark very justly that "the circumstances of the inhabitants of a great portion of the continent at this time, arising from the distress occasioned by its being the seat of war, must have strongly resembled those of the people of Ireland during the late scarcity of provisions. At a later period, in 1817, after a failure of the crops, epidemic fever existed in the southern parts of Italy. . . . . From the same authority (Dr. Pockles) we learned that in the early part of 1817, scarcity of food was so great in Germany that many died of hunger; but no epidemic fever existed there at that time. It had prevailed in that country three years previously, and did not then originate in scarcity of provisions, but was traced to the miserable remnant of the French army which entered that country after its overthrow in Russia. From the facts here adduced, it follows incontrovertibly that during the times of its increase in Ireland fever was very prevalent in most parts of the continent, and that the circumstances which caused it to spread epidemically were not peculiar to this island. . . . . . But whatever may have been the causes which have rendered the disease more than usually frequent during the last nine or ten years, no distinct evidence has been obtained of its introduction from the continent; and an inspection of the preceding table (vol. i. p. 49) points out that the rapid increase of the disease depended on general causes, operating on most parts of the country at the same time. For we find that it commenced in places situated most distant from each other in different parts of Munster and Ulster, at the end of 1816 or beginning of 1817; and making the proper allowance for the difficulty of determining when fever became epidemical in places, which are always infested by the disease, we must admit that the periods of its manifest increase were nearly coincident. In fact, the scarcity of provisions combined with want of employment, whatsoever their mode of operating may have been, appears as the main cause of the spreading of fever epidemically through this country; although it must also be acknowledged that the simultaneous increase of this disease in Ireland and on the continent, leads to the inference that whatever may have been its origin an epidemic constitution prevailed over a great part of Europe during a series of past years. . . . . With respect to the time of its greatest prevalence in each of the four provinces it is not easy to decide. In Munster, it appears to have been most prevalent in the summer of 1818, and in Connaught about the same time, whilst the other provinces, where its commencement was latest, the time of its greatest prevalence was referred in Leinster generally to the autumn of 1817, and in Ulster to the winter of that year. In the principal cities, Dublin, Cork, and Limerick, it was most prevalent in the summer and autumn of 1818."*

* Barker and Cheyne on Fever, pp. 25-107.

[To be completed in the next Number.]

We have thus made very copious extracts from Drs. Barker and Cheyne's valuable record of the epidemic fever in Ireland; as it contains a greater amount of facts and observations respecting this disease than any work that we have consulted. Indeed, when we consider that about one fourth, or a million and a half, of the population of Ireland were affected with fever, during the two years that it prevailed, and that accurate communications were received from respectable physicians residing in all the provinces, and that these have been admirably concentrated and illustrated by the authors, it must be considered one of the most important as well as interesting descriptions of the rise, progress, and decline of this disease.

Dr. Adams states that "during the winter of scarcity in 1799 and 1800, fever from infectious atmosphere was so general as to excite us to imitate the example of those manufacturing towns which are never free from the disease, and a fever-house was established in London."* Dr. Bateman remarks that "deficiency of nutriment is the principal source of epidemic fever, and that the circumstances just alluded to (improvement in all the arts of life), operate only as accessories in fostering and multiplying it will scarcely admit of dispute. . . . . The last epidemic which occurred in London followed a scarcity of two successive years (1799 and 1800); and it was during the prevalence of this fever that the necessity for establishing a House of Recovery became manifest. . . . Whether the epidemic of 1817 has been really much more extensive than the former, I am unable to determine. . . . . It might have been expected, indeed, that the present epidemic would exceed the last in the extent of its course, since it occurred at a period of unparalleled distress among the labouring poor; when the loss of employment, occasioned by the termination of the war and the general suspension of the manufactures, concurred with the failing harvest of 1816 to increase the difficulties of procuring subsistence."† Dr. Tweedie observes that "it is an undeniable fact founded on the experience of many epidemics, that there are certain circumstances which render the system peculiarly predisposed to the action of febrific causes; and the connexion of scarcity and privation with the occurrence of fever among the lower classes of the community, has been so often verified by the experience of epidemics, as now to be received as a general axiom."‡ The same author also makes the following observations on the influence of the temperature and moisture of the atmosphere: "Though fever can scarcely be said to have prevailed extensively, or to adopt the common phrase, to have been epidemic in London since 1820, yet the diminution of autumnal fevers, for the last two seasons, proves decidedly how much some unknown condition of the atmosphere influences its prevalence. This condition is intimately connected with the combined effects of heat and moisture; hence cold and wet summers are always remarked to be comparatively healthy, while disorders of the bowels in such seasons are seldom observed. The number of patients admitted into the Fever Hospital in the autumn months of the last three years establish this principle. In August, September, and October, 1827, there were admitted 205; in

* Adam's Inquiry into the Laws of Epidemics, p. 30.
† Bateman on Contagious Fever, pp. 4 to 11.
‡ Tweedie's Clinical Illustrations of Fever, p. 78.

the same months of 1828, the numbers were 170; in the autumn of 1829, only 94 were received. The cause of this progressive diminution is undoubtedly to be traced to the cold wet summers of the last two seasons."* An opinion, exactly opposed to that of Dr. Tweedie, is given by Dr. Armstrong. He states that "in England typhus is evidently favoured by a low temperature, being most prevalent in the cold seasons of winter and spring, generally abating or disappearing as the heat of summer advances, and often prevailing to a considerable degree in cold wet autumns."† Dr. Alison makes the following statement respecting the cause of the epidemic fever which prevailed in Edinburgh during the years 1826-7: "The chief cause of the unusually great and rapid extension of fever during last winter was no doubt the very distressed condition of a great part of the lower order of inhabitants, in consequence of the diminished expenditure of the higher ranks, and particularly of the failure of many speculations in building, which had given employment to great numbers of masons, joiners, plasterers, and labourers. . . . . A very great number of the patients received into the hospitals in fever belonged to families of which the working members had been out of employment for periods varying from six weeks to six months; and Edinburgh has furnished but too many opportunities, both recently and formerly, for observing that it is among such distressed families that fever spreads most rapidly and extensively."‡

Dr. Cowan attributes the increase of fever in Glasgow, which has steadily been going on since 1816, principally "to the total want of cleanliness among the lower orders of the community, to the absence of ventilation in the more densely peopled districts, and to the accumulation for weeks or months together of filth of every description in our public and private dunghills; to the over-crowded state of the lodging-houses resorted to by the lowest classes; and to many other circumstances unnecessary to mention."§ The same author, in another part of his statistics, illustrates the causes which tend to render fever epidemic; and he makes the following observations: "From the close of 1836, one of those periodical depressions in trade, arising from the state of our monetary system, has visited this city, and deprived a large proportion of the population of the means of subsistence. From the existence of secret combinations among the working classes in various departments of trade, but especially among the cotton spinners, and the 'strikes' which resulted from these combinations, a very large proportion of the inhabitants, in addition to those already suffering from the state of the money market, were suddenly deprived of employment, and consequently of the means of procuring food. The high price of coal was the means of diminishing the hours of labour, and consequently the amount of wages, in numerous factories, and placed fuel beyond the reach of the lower classes for domestic purposes. And in addition to these sources of misery, the average prices of grain were much higher during 1837 than they had been for some years previously.|| . . . A reference to the tables of the state of the weather given in the preceding part of

* Tweedie's Clinical Illustrations of Fever, p. 80.
† Armstrong on Typhus Fever, p. 8.
‡ Edinburgh Medical and Surgical Journal, vol. xxviii. p. 236.
§ Cowan's Vital Statistics of Glasgow, p. 13. || Ibid. p. 33.

this paper, will show the quantity of rain which fell, monthly, during the period of my attendance on the Fever Hospital, and the average temperature indicated by Fahrenheit's thermometer. From these it appears that the quantity of rain was much above the average, while the temperature of almost every month was lower than that of the previous year; and while the mean heat of Glasgow is 47° 75, the mean heat of 1835 was 46° 58, and that of 1836 only 44° 52.* . . . .

| | |
|---|---|
| "The number of fever patients treated in Hospital in 1837, was . . . | 5387 |
| " " 1836 " . . . | 3125 |
| Being an increase in 1837 of . . . . | 2262 |
| The number of fever patients treated by the district surgeons in their own houses in 1837, was . . . . . . | 2320 |
| Ditto Ditto, in 1836, was . . . | 716 |
| Being an increase of . . | 1604" |

The above table gives a very inadequate idea of the comparative frequency of fever in 1836 and 1837. During 1836, till the month of December, every applicant for admission was received into the hospital: while in 1837, seldom a day passed without numerous applicants being refused admission for want of room; and many were deterred from applying for admission from a knowledge of the over-crowded state of the wards.† Dr. Cowan calculates the number of fever cases in Glasgow, during the years referred to, as follows:

| | |
|---|---|
| "In 1835 . . . . | 6·180 |
| 1836 . . . . . | 10·092 |
| 1837 . . . . | 21·800 |
| | 38·072‡" |

After having given a table of the deaths from fever during each month of the years 1836 and 1837, the same author makes the following deductions from it. " Many interesting observations may be drawn from this table. It shows the slow progress of an epidemic disease when trade is prosperous, compared with what occurs in seasons of distress. Up to November, 1836, the period at which the commercial embarrassments were felt, the mortality from fever had not been rapidly increasing. In November it was just about double what it had been in January preceding, the number of deaths being 45 in January, and 89 in November. The moment, however, the effects of the stagnation in trade extended to the working classes, the mortality increased with fearful rapidity, aided, no doubt, by the season of the year, the high price of grain, and the scarcity or high price of fuel. . . . . The table also marks the period at which the epidemic reached its maximum amount of mortality, namely, in the second quarter of 1837, and in the month of May in that quarter, being the month succeeding that in which the strike of the cotton spinners took place, by which 8000 individuals were thrown out of employment."§ The total quantity of rain, according to Dr. Cowan, which fell during 1837, as ascertained at the University of Glasgow, was 26·629 inches; and the mean temperature for that year was 46° 31. ||

* Cowan's Vital Statistics of Glasgow, p. 16. † Ib. p. 37. ‡ Ib. p. 38.

§ Ib. p. 39. || Ib. p. 35.

We have constructed the following tables from the registers of the weather and the table of deaths from fever in Glasgow, as given in Dr. Cowan's work, in order to show, that though the increased quantity of rain during the years 1836 and 1837 was influential in the diffusion of fever, yet that it had less effect in spreading the epidemic than other causes; for during the year 1837, at which time it had reached its maximum, the total quantity of rain that fell was less than during 1836, while the average temperature of these two years did not materially differ from each other.

| 1836. | Deaths from Fever. | Mean Temperature. | Quantity of Rain. |
|---|---|---|---|
| January . . . . | 45 | 37°84 | 3·868 |
| February. . . . | 27 | 35 39 | 0·732 |
| March . . . . . | 57 | 38 60 | 2·375 |
| April. . . . . | 64 | 42 05 | 1·098 |
| May . . . . . | 67 | 51 04 | 0·173 |
| June . . . . . | 71 | 55 19 | 1·812 |
| July . . . . . | 61 | 54 38 | 4·536 |
| August . . . . | 82 | 53 17 | 5·317 |
| September . . . | 56 | 47 37 | 2·134 |
| October . . . | 89 | 42 89 | 4·988 |
| November . . . | 89 | 38 11 | 2·004 |
| December . . . | 133 | 38 27 | 2·673 |
| | * 841 Total. | † 44·52 Average | ‡ 31·710 Total. |
| 1837. | | | |
| January . . . . | 201 | 36°13 | 1·956 |
| February . . . | 138 | 40 55 | 2·674 |
| March . . . . . . | 224 | 34 39 | 1·500 |
| April . . . . . . | 202 | 38 28 | 1·646 |
| May . . . . . . | 233 | 48 21 | 1·857 |
| June . . . . . . | 199 | 56 89 | 2·241 |
| July . . . . . | 194 | 60 80 | 3·322 |
| August . . . . | 172 | 56 90 | 2·610 |
| September . . . | 126 | 52 99 | 1·570 |
| October . . . . | 149 | 48 92 | 2·997 |
| November . . . | 147 | 39 68 | 2·293 |
| December . . . | 195 | 41 95 | 1·963 |
| | § 2180 Total. | ‖ 46·31 Average | ‖ 26·629 Total. |

The number of deaths from fever in a given period is not an exact criterion of the number of persons affected; for the intensity of this disease varies considerably at different seasons of the year; but as the number of deaths which occurred in each month of 1836 and 1837 respectively, are given, the relative mortality will give a pretty near approximation to the relative number affected.

Dr. Arthur Thomson, in his Statistics of Fever, has given two tables, which show the influence of the seasons on the prevalence of fever; and as his conclusions are drawn from a large number of cases, they are well adapted for illustrating this part of the subject.

* Cowan's Statistics of Glasgow, p. 38. † Ib. p. 5. ‡ Ib. p. 4. § Ib. p. 38. ‖ Ib. p. 35.

"TABLE XII. *Showing the maximum, minimum, and mean temperature in Great Britain during each month, from the observations of about thirty years, together with the average monthly quantity of rain in inches from thirty-four years' observation (from* 1797 *to* 1830.) *

| Months. | Mean temperature. | Average quantity of rain in inches. |
|---|---|---|
| January . . . . . . . . | 36° | 1·90 |
| February . . . . . . | 38 | 1·49 |
| March . . . . . . . . | 43 9 | 1·39 |
| April . . . . . . . . . | 49 9 | 1·84 |
| May . . . . . . . . . | 54 | 2·00 |
| June . . . . . . . . . | 58 7 | 1·94 |
| July . . . . . . . . . | 61 | 2·55 |
| August . . . . . . . | 61 | 2·15 |
| September . . . . . . | 57 | 2·29 |
| October . . . . . . . | 48 | 2·41 |
| November . . . . . . | 42 | 2·79 |
| December . . . . . . | 39 | 2·58 "† |

From the above it appears that July and August are the months during which the average temperature is greatest, and that the quantity of rain falling during the last six months of the year is considerably more abundant than during the first six. The following table this author has "compiled from materials selected indiscriminately from all the reports which he could obtain, showing the number of fever cases admitted into the various hospitals in Great Britain and Ireland; but he is chiefly indebted to Drs. Barker and Cheyne's account of the epidemic fever which prevailed in Ireland in 1817-18-19."

"TABLE XIII. *Showing that of* 51,944 *cases of fever admitted into different hospitals in Great Britain and Ireland, the number and relative ratio of admissions in each month were as follow:*

| Months. | No. of Cases admitted. | Relative ratio of admissions per cent. |
|---|---|---|
| January . . | 2895 | 5·6 |
| February . . | 2825 | 5·4 |
| March . . | 3152 | 6·1 |
| April . . . | 3374 | 6·5 |
| May . . . | 3990 | 7·6 |
| June . . . | 4365 | 8·3 |
| July . . . | 4999 | 9·6 |
| August . . | 5261 | 10·1 |
| September . | 5046 | 9·7 |
| October . . | 5624 | 10·8 |
| November . | 5054 | 9·7 |
| December . . | 5359 | 10·6 |
| | Total 51,944 | 100·0 |

"It appears from this table that the greatest number of fever cases were admitted into the different hospitals, during the last six months of the year, or from July to December. And the number of cases admitted

* The maximum and minimum temperature is omitted.
† Howard on Climate of London, 2d Edit. Vol. i. p. 136.

from January to June are few, compared with the admissions from July to December." *

In order to compare the number of admissions in each month, with its mean temperature and average quantity of rain, we shall construct a table out of the two that have just now been quoted; which will show the number of admissions, the mean temperature, and the average quantity of rain for each month.

| Months. | No. of Cases admitted. | Mean Temperature | Average quantity of rain in inches. |
|---|---|---|---|
| January . | 2895 | 36° | 1·90 |
| February . . | 2825 | 38 | 1·49 |
| March . . | 3152 | 43 9 | 1·39 |
| April . . . | 3374 | 49 9 | 1·84 |
| May . . | 3990 | 54 | 2·00 |
| June . . . | 4365 | 58 7 | 1·94 |
| July . . | 4999 | 61 | 2·55 |
| August . . | 5261 | 61 | 2·15 |
| September . | 5046 | 57 | 2·29 |
| October . . | 5624 | 48 | 2·41 |
| November . | 5054 | 42 | 2·79 |
| December . | 5359 | 39 | 2·58 |
| | 51·944 | | |

This table shows that the greatest number of fever cases were admitted into the various hospitals from July to December, or during the last six months of the year; and that during this period the average quantity of rain which falls is much greater than during the first six months of the year. If we compare any one month of the last six with any one month of the first, there will be found a similar difference. The same table also shows that the temperature may vary considerably during a similar prevalence of fever, and that nearly the same temperature may prevail with a great variation in the number of cases. Thus, in August the number of cases is 5261, and in December the number of cases is 5359, being a difference only of 98; but the mean temperature of the first-mentioned month is 61°; while that of December is only 39°; the quantity of rain, however, in both of these months is above the average. In March the mean temperature is 43° 9, and the number of cases 3152; while in November the mean temperature is 42°, and the number of cases 5054; but the quantity of rain in March is 1° 39, while in November it is 2°79, being double the amount of that which falls in the first-mentioned month. In February the mean temperature is 38°, and the number of cases 2825; while in December the mean temperature is 39°, aud the number of cases 5359; but the quantity of rain in the first of these months is 1·49 inches, while in December it is 2·58 inches.

The conclusions which may be drawn from this table, are, that in all the months in which the quantity of rain is above the average, fever prevails to a greater extent than in those months in which it is below this point. It does not appear, however, from it that the average range of temperature of this climate has much influence on the prevalence of

* Edinburgh Medical and Surgical Journal. July, 1838. p. 100.

fever; for if moisture be present, it may prevail to about the same extent, when the average temperature is 61°, as in August, or when it is 39°, as in December.

The diffusion of fever is thus generally connected with humidity of the atmosphere; yet certainly there are other causes of a more influential kind that are also in operation. This is well exemplified by the two tables (page 52), which show the prevalence of fever and the corresponding weather in Glasgow during the years 1836 and 1837. Thus, although in both of these years the quantity of rain was greater than the average, and in the first of them greater than in the second, yet the number affected with fever during 1836 amounted only to about the half of those that were seized during 1837. The increased prevalence of the epidemic during 1837, must, to a very considerable extent, have depended upon the scarcity of provisions, want of fuel, &c., and their concomitants, filth, &c., which followed the commercial embarrassments of that year.

*An accumulation of persons not previously affected tends to diffuse typhus.* There is also another important point connected with the history of eruptive typhus, which has seldom been taken into calculation in attempting to account for its diffusion, namely, that it does not often attack the same person more than once during his life. Now if this be admitted,—and we have endeavoured to show at page 16, the analogy between typhus and other exanthematous fevers in this particular, but even though M. Hildenbrand's modified view only be granted, namely, that it secures the person who has been affected only for some years—it follows:

1st. That after an epidemic fever has prevailed for some time, it must cease after the lapse of a particular period, from deficiency of *material* to act upon; and the history of almost every pestilence of this kind, shows that it rarely exceeds two years in duration, even in a large city.

2d. That, though fever may constantly exist in a large town, in a minimum proportion, varying in numbers according to the habits of the people, those who have never laboured under the disease are gradually accumulating; and that when the state of the atmosphere, as to humidity, and the scarcity of provisions, with their consequents, filth and deficient ventilation, are concurrent with this accumulation of susceptible individuals, that fever has rarely failed to spread among the community. And if the population of any large city be increasing very rapidly, such as that of Glasgow, at the rate of ten thousand persons annually, the number of susceptible individuals will be accumulated in a few years to an amount, sufficient for the existence of an extensive epidemic.

3d. That a severe epidemic fever of one or two years' duration is never succeeded by another until several years have elapsed.

A very important enquiry may be deduced from the foregoing statements. Can the very rapid increase of the population of Glasgow account, to a greater or less extent, for its being visited for a series of past years with more frequently recurring epidemics of fever than any other city of Great Britain, similar in size and population? Can the influx of several thousands of unprotected individuals from the country every year afford any explanation of the occurrence; just as there is always a great mortality from smallpox in Glasgow, from the influx of unvaccinated Highlanders, while in many other cities of the kingdom this disease is comparatively rare? There can be no doubt that the influx of so many strangers to this city must have a powerful effect in increasing the number of fever patients; but certainly the filthy and irregular habits of its working population are equally operative as predisponents to contagion. Is there then any prophylactic measure which may either ward off or diminish the extent of an epidemic diffusion of fever in a large city; or is this beyond the control of human means and calculations? We think not; although we do not entertain the notion that fever will ever be completely extinguished in any large manufacturing town; or that the spread of it, epidemically, can be checked *in limine*, when the concurrent circumstances are favorable for its propagation; but certainly much might be done to lessen the intensity of the evil. It has already been shown that filth and deficient ventilation tend much to spread the contagion of typhus, being almost constant concomitants; and that while it generally affects the whole members, or the large proportion of a family, among the lower orders, it rarely spreads in this manner among the better classes of society, who attend more to cleanliness and ventilation. It is quite obvious that an amelioration of the physical condition of the lower orders, in these particulars, would, in proportion as this was effected, diminish their chances of catching the contagion; which would not only operate in lessening directly its diffusion, but, by reducing the number of its sources, must tend to lessen the actual quantity of this principle that might be generated in a given time.

But can this amelioration be effected to any appreciable extent; or if effected, could it be maintained for any length of time? We fear that little permanent amelioration could be effected without a legislative enactment; for though our philanthropists are very active in their charities during the prevalence of an epidemic, it no sooner subsides than they relapse into a comparative quiescence, and our working population into their former habits of filth and intemperance. And the evil will continue to assail us so long as our cities contain so many narrow and filthy lanes, so long as the houses situated there are little better than dens or hovels, so long as dunghills and other nuisances are allowed to accumulate in their vicinity, so long as these hovels are crowded with inmates, and so long as there is so much poverty and destitution. Why, then, should we not have a legislative enactment that would level these hovels to the ground, that would regulate the width of every street, that would regulate the ventilation of every dwelling-house, that would prevent the lodging-houses of the poor from being crowded with human beings, and that would provide for their destitution? It may be said, that this would interfere too much with the liberty of the subject, and no doubt it would be vehemently opposed by many interested persons. In place, however, of being an infringement on the liberty of the subject, it might rather be designated an attempt to prevent the improper liberties of the subject; for what right, moral or constitutional, has any man to form streets, construct houses, and crowd them with human beings, so as to deteriorate health and shorten life because he finds it profitable to do so? As well ought the law to tolerate the sale of unwholesome food, because it might be profitable to the retailer of it.

### CHAPTER III.

### CIRCUMSTANCES WHICH TEND TO RENDER FEVERS COMMUNICABLE FROM ONE PERSON TO ANOTHER.

It is quite obvious, if the doctrine of contagion be admitted, that all those circumstances which favour the diffusion of fevers tend also to render them communicable from one person to another; it is therefore necessary to include them in the following arrangement, although it seems only necessary to illustrate one of them a little farther, namely, the influence of filth and deficient ventilation.

Circumstances which tend to render fevers communicable:

1. Humidity of the atmosphere.
2. Scarcity of provisions, &c.
3. No previous affection with typhus.
4. Filth and deficient ventilation.
5. Age.
6. Acclimatization.
7. Idiosyncrasy of constitution.

Alleged circumstances which tend to render fevers communicable:

1. Weakness of constitution.
2. Greater susceptibility of females.
3. Depressing passions.
4. Intemperance.

Alleged exemptions from fever:

1. From trade or occupation.
2. From chronic diseases.

These different points shall be considered in the following part of the essay, though not exactly in the order enumerated, as this might derange the general connexion of the observations.

*Influence of filth and deficient ventilation.* In a previous part of the essay we entered into the consideration of filth and deficient ventilation as tending very powerfully to spread the contagion of typhus; and showed that, where it was concentrated, as in crowded hospitals, or in the small and ill-ventilated houses of the lower classes, it rarely failed to be communicated to the unprotected attendants or inmates of a family. Filthiness of personal habits, however, although it tends to render it more communicable, as we shall endeavour to show from the statistics of the Glasgow Fever Hospital, does not seem to act so powerfully in this respect as deficient ventilation, which by concentrating the contagion may render its operation on the system more certain. In proof of which we may quote the various attendants of our fever hospitals, who are generally very attentive to cleanliness in their persons, and yet, if unprotected, are almost uniformly affected with fever during some period of their attendance, if the wards be in a crowded state. This fact, and the more frequent exemption of the attendants when the wards are moderately filled and well ventilated, seem to prove that contact with the patient is not so essential for the communication of the disease, as being surrounded by an atmosphere highly impregnated with the contagious miasmata. And there are many instances where students have been affected with fever after visiting the wards of an hospital, without having come into contact with the patients or their bed-clothes. There can be no doubt, however, that simple contact of a typhus patient, or of clothes that have been attached to him in any shape, may communicate the disease without the aid of even a partial impregnation of the atmosphere with contagious effluvia, and where the most perfect ventilation has been maintained.

We are at present unacquainted with the channel by which the contagion of typhus most generally enters the body; and though the opinion be generally entertained that the lungs are the organs through which it passes into the system, yet it is equally probable and consistent with analogy and facts to believe, that the skin is, at least, as important as a medium of communication. There are many animal poisons that operate on the system through the skin very powerfully, and yet have little effect when applied to the mucous membrane of the stomach or intestines; now though, in the one case, the poison operated with be ponderable and be applied to the mucous surface of the stomach and bowels, while, in the other case, the contagion of typhus is imponderable, and is applied to the mucous surface of the bronchial tubes, yet, in the absence of direct experiment, this analogy is entitled to some consideration.

The following tables will tend to show that filthiness in personal habits is very frequently connected with the production of typhus, and it includes all the cases of fever that were admitted into the Glasgow Fever Hospital from May 1st to November 1st, 1839, in whom their state as to cleanliness or filthiness was ascertained:*

| | FILTHY. | | CLEAN. | |
|---|---|---|---|---|
| | Males. | Females. | Males. | Females. |
| Scotch.. .............. ...... | 92 | 81 | 64 | 93 |
| Irish ..... .............. ... .. | 88 | 70 | 60 | 47 |
| English ...................... | 4 | 2 | 3 | 3 |
| West Indies and North America ................ | 3 | | | 1 |
| | 187 | 153 | 127 | 144 |
| | Total filthy...340 cases. | | Total clean...271 cases. | |

The following table shows the number of cases that were filthy and those that were clean in typhus characterized by the eruption, and also the proportions, regarding this point, which were ascertained in febricula:

| | FILTHY. | | CLEAN. | |
|---|---|---|---|---|
| | Males. | Females. | Males. | Females. |
| Eruptive typhus | 133 | 112 | 73 | 77 |
| Febricula......... | 6 | 8 | 19 | 15 |
| Total no. of cases of febricula, filthy......14 | | | Total no. of cases of febricula, clean...34 | |
| — of typhus, — ......245 | | | — of typhus, — ...150 | |

* The reports, respecting the clean or filthy state of the patients admitted, were taken by the barber of the Fever Hospital, and afterwards transferred by the author, along with other statistical facts, into his own journal.

These two tables show that among 611 cases admitted as continued fever, there were 340 filthy and 271 clean, or about 55 per cent. filthy; that among 395 cases of eruptive typhus, there were 245 filthy and 150 clean, or about 62 per cent. filthy; and that, among 48 cases of febricula, there were 14 filthy and 34 clean, or about 29 per cent. filthy. The following deductions may be drawn from these facts. 1. That the proportion of filthy persons is greater than that of the clean among the whole number of cases admitted, and including not only typhus, but bronchitis, febricula, pneumonia, and several other affections which are specified in a table already given. 2. That among the eruptive or decided cases of typhus, the proportion of the filthy to the clean is still greater than what exists among the whole number of cases. 3. That among the cases of febricula, the proportion of filthy persons is only 29 per cent., while in eruptive typhus it is 62 per cent. 4. That, as the proportion of filthy persons in the whole number of cases is less than in those affected with eruptive typhus, it is fair to infer that this is owing to an admixture, with the latter, of febriculous, bronchitic cases, &c., since it has been shown that filthiness is much less frequently a concomitant of febricula, &c., than it is of eruptive typhus.

We are entirely ignorant of the nature of those substances which absorb the typhus contagion with most facility; but, as filth is very frequently a concomitant of its ready communicability, it may be assumed that either the clothes or the deposits on the skin of filthy persons have a tendency to absorb the contagion and to retain it until the system become affected. We know that certain gases, and even odours and fetid effluvia, are absorbed more readily by some substances than by others; and though we are only warranted to assume from this analogy, that typhus contagion is very probably regulated by a similar law, yet, on the other hand, if want of cleanliness facilitate the operation of contagion on the system, it is not possible to explain this effect on the principle of pulmonary inhalation, while the theory of cutaneous absorption is not opposed by any fact or analogy. If this view be adopted, it will obviously lead to a prophylactic measure of considerable importance, namely, the daily and thorough ablution of the skin, and the frequent changing of the wearing apparel, for it is not probable that the contagion will be absorbed immediately after its application to the clothes or skin of the person who has been exposed to it; and by the daily ablution of the whole body, it may be removed before this can occur.

*Influence of idiosyncrasy of constitution.* The contagion of typhus is not communicable to all persons with the same facility. Some individuals are infected after the first exposure, while others may be exposed for weeks, or even many months, almost constantly, before they are attacked. It may be said, however, that in this last case the contagion has remained for a longer time latent than in the former. There is no very precise evidence existing as to this point, and the opinions entertained by many authors are often conjectural. Dr. Bancroft states as follows: " It results, therefore, from this statement, that among the ninety-nine orderlies and nurses who had probably not been exposed to the contagion before their attendance on the sick commenced, the earliest attack was on the 13th day, and the latest on the 68th; but these returns were made up about the 20th of April, and it appears that some who had escaped till that time were afterwards attacked; and therefore, though there may be reason to conclude that febrile contagion does not remain inactive so long after being received into the body as marsh miasmata, I see none for believing that an interval of five or six months may not sometimes elapse before the actual production of fever by it."* Dr. Perry is of opinion, that "the earliest period of the disease making its appearance after exposure to contagion is eight days, more frequently fourteen, and sometimes so long as two months."† What the circumstances are which render some persons, who enjoy good health, are well fed and cleanly in their personal habits, more susceptible of contagion at one period than at another are totally unknown; but we are in the same state of ignorance as to the reason why scarlet fever, &c., may be caught at one period and not at another, and why vaccination frequently succeeds at last after five or six unsuccessful trials. Again, a certain proportion of persons appear not to be susceptible of the disease. Dr. Perry is the only author that we are acquainted with that enumerates the proportions of susceptible and non-susceptible individuals. His 10th proposition is the following: "That between the ages of seven and fifty, sixteen out of twenty are susceptible of being affected with contagious typhus, if exposed to the contagion, and not protected by having previously had the disease."‡

That there is a certain proportion of individuals who are not susceptible of typhus contagion there can be no doubt, for there are many medical practitioners and nurses of fever-hospitals who have never laboured under the disease, although they have been exposed to its influence for many years, but there is no proof that in the present state of our fever statistics we can define the proportion of unsusceptible persons. It is a common opinion that constant exposure lessens the susceptibility to fever; but this, in the present state of our knowledge, can only be considered as a probable hypothesis.

*Influence of sex.* Hildenbrand is of opinion that delicate men, who have a fine skin and feeble bodies, are most subject to contagion; while, on the contrary, those that are robust, plethoric, vigorous, and well nourished, more seldom contract it. These opinions are entertained by several writers on fever; and for a similar reason, it is sometimes concluded that females are more subject to this disease than males. It is natural for an author, who advocates the absorption of contagion by the skin, such as Hildenbrand, to infer that a fine skin, like that of the female, will absorb more readily than one which is coarse; and, although this theory be supported by the statistics of some hospitals, it is opposed by those of others. The number of admissions into the Glasgow Fever Hospital during the year 1836 were 1116 males and 1141 females,|| which is only a small excess of females; but if the excess of the female over the male population of Glasgow be taken into the account as about one sixth, the proportion of males that have been affected with fever will be plus instead of minus. In the same institution were admitted, from May 1st to November 1st, 1839, 270 males and 276 females, classified under typhus.

* Bancroft on Yellow Fever, &c. p. 516.
† Edinb. Med. and Surgical Journal, vol. xlv. p. 69.
‡ Ibid. p. 67.
|| Cowan's Vital Statistics, p. 19.

Into the Cork-street Fever Hospital, Dublin, from 5th January, 1817, to 30th April, 1818, there were admitted 2883 males and 2849 females, which is a small excess of males.* Again, in other hospitals, there has occurred an excess of females. There were admitted into the Waterford Hospital 1277 males and 1452 females,† into the London Fever Hospital 1229 males and 1308 females,‡ into the Limerick Fever Hospital 1332 males and 1895 females, being a large excess of females,§ and into the Edinburgh Royal Infirmary 962 males and 1075 females.|| The facts which have been hitherto published regarding the susceptibility of the different sexes to fever are not yet sufficiently extended to warrant us drawing any certain conclusion from them; but certainly it does not appear to be established by satisfactory evidence, that the one sex is more liable to the disease than the other; and, where this does occur in any particular place, that it cannot be accounted for by the general excess of female population in large cities, or by other circumstances connected with their history. Drs. Barker and Cheyne remark, that "in Dublin, when the epidemic had completely established itself, the males admitted to hospital were most numerous, but in its progress the admissions of females exceeded those of males.... As to the comparative frequency of fever in the male and female sex in the country at large, we can form no decisive opinion, the answers to our enquiries on that head not having been perfectly satisfactory."¶

Although the comparative frequency of fever among the sexes has not been accurately determined, it has been proved satisfactorily by the statistics of almost every large hospital, that a larger per cent. of males than of female patients die of the disease; and it is proved by the Glasgow Mortality Bills, that a much greater number of the male than of the female population of that city are carried off by it. Thus, in Glasgow, during the year 1836, 465 males and 376 females died of fever; during 1837, 1187 males and 993 females; and during 1838, 439 males and 377 females.** If the average mortality of each sex could be accurately ascertained, this large amount of deaths might be made available for determining the liability of the different sexes to fever, by the same method of approximation which Dr. Cowan has adopted in calculating the amount of fever in Glasgow during the years 1836 and 1837;†† but as the proportionate mortality of the different sexes is not the same during every season, and as it may not be the same among those treated at home as in those treated in hospitals, this method, although well adapted for giving a general approximation, is not well calculated for determining a nice question of this kind.

*Influence of delicacy, or weakness of constitution.* We have already remarked, that it is a prevalent opinion among medical men, that persons naturally weak and delicate are more liable to fever than those

* Barker and Cheyne on Fever, vol. i. p. 91. † Ib. p. 193.

‡ Dr. S. Smith's Treatise on Fever, p. 432.

§ Dr. Geary's Report, Dublin Journ. of Med. Science, vol. xii. p. 10.

|| Edinb. Med. and Surgical Journal, Oct. 1839, p. 448.

¶ Barker and Cheyne on Fever, pp. 89-90.

** Glasgow Bills of Mortality for 1836, 1837, and 1838.

†† Dr. Cowan estimated the proportion of the whole mortality in 1837 as 1 in every 10 patients; and to determine the amount of cases, multiplied the whole number of deaths, which were 2180 by 10 = 21·800.

who are healthy and vigorous. This opinion seems to be as little capable of proof as the preceding one regarding the greater liability of females to the disease. We are not, however, in possession of much evidence, and none statistical, so far as we are aware, regarding this point, beyond the loose and general observations of authors.

We have kept a record of the physical habit of the patients admitted into the Glasgow Fever Hospital from May 1st to November 1st, 1839, and the following were the divisions adopted :

1. Moderate, by which is meant a person having an ordinary quantity of muscle and cellular substance.
2. Full or plethoric, having an extra quantity of adipose texture or of blood.
3. Muscular.
4. Spare.
5. Emaciated or unhealthy in appearance.

| | Males. | Females. | Total. |
|---|---|---|---|
| Moderate | 116 | 93 | 209 |
| Full or plethoric | 28 | 73 | 101 |
| Muscular | 44 | ... | 44 |
| Spare | 24 | 41 | 65 |
| Unhealthy or emaciated | 2 | 8 | 10 |
| | | | 429 |

The whole of these 429 cases were characterized by the typhoid eruption, and will therefore be considered as decided cases of typhus. It appears from this table, that there were only 10 cases in an emaciated or unhealthy condition; and almost all of them, as far as could be ascertained, were engaged in their ordinary occupations at the time of their seizure. The spare and unhealthy, when added together, only form about 17 per cent. of the whole number.

*Influence of chronic diseases.* The evidence, such as we have collected from the previous history of patients admitted into the Glasgow Fever Hospital; and from post-mortem examinations, seems to prove that persons affected with any particular chronic disease of the chest or belly, are very rarely affected with typhus fever. Hildenbrand states that phthisical persons are very rarely affected with typhus fever; and that, out of many hundred cases of this disease that he has treated, not one instance of a phthisical person has occurred. We have heard the same opinion expressed by several physicians of extensive hospital experience, and that they have scarcely ever met with a case of tubercles in a person who has died of eruptive typhus.

This opinion we can nearly confirm from our own experience, for out of more than 100 post-mortem inspections we have met with only three cases; and the number of tubercles in each did not exceed three, which were small and only partially softened.

*Influence of fear and the depressing passions.* The influence of fear and the depressing passions has also been considered as very powerful in predisposing persons to be affected with typhus contagion. There can be no doubt that fear has a tendency to produce a temporary depression of the physical powers; but, as has been already shown, there is no proof that persons of a naturally spare or weak habit of body, who are generally very sensitive, are more liable to fever than those of an ordinary constitution, this opinion must also be considered hypothetical. Indeed, the facts, as far as our enquiries have enabled us to judge, seem to prove that the apprehension of fever, more particularly when it is not epidemic, is very rarely felt until the person is actually seized with the disease; for some cannot recollect of a single circumstance by which they could be exposed to contagion; and a considerable number of those who had undoubtedly been exposed to it, were only made aware of the fact when it had been elicited by cross-examination. We are quite aware that cases may be brought forward, of sensitive individuals who have been seized with fever soon after visiting a person labouring under the disease; but as this fact can be opposed with at least an equal number of persons who were destitute of fear, and yet caught it after an exposure to contagion, no conclusion whatever can be drawn from them. It must be observed, however, that though there is no proof that persons who are naturally weak in body or of a sensitive disposition are more susceptible of fever than those who are naturally vigorous and robust, yet that, during famine or commercial distress, poverty by depressing the mind and lowering the physical status from insufficient aliment, does powerfully predispose a community to become affected with fever. This has been already shown in a former part of the essay; and has been again alluded to, in order that the distinction might be made between an individual of naturally weak mental and physical stamina, and one who has been reduced to that state by deficient nutriment.

*Influence of intemperance.* It is a question of vital importance to the inhabitants of large towns, whether intemperance predisposes those who indulge in it to be affected with fever. A solution of this point in a satisfactory manner cannot, we are afraid, be made from our present data; for no statistics regarding it have been published. Indeed, it is sometimes very difficult, even after the most careful enquiries, to find out the habits of patients who are sent to an hospital; for most of them are ashamed to acknowledge intemperance when it does exist, and those who admit that they indulge a little are sometimes more abstemious, in point of quantity, than those who deny any indulgence whatever. The ascertaining of such habits, accurately, is in many cases impossible, and the evidence must be viewed principally as an approximation to the truth. At the same time, this approximation may be often rendered very convincing, by sifting the answers of the patients, by an attentive examination of their appearance, and by the evidence of friends; and occasionally conclusions confirmatory of the opinion formed may be drawn from their trade or occupation; for it is well known, that in some occupations the majority of the workmen are addicted to excessive drinking. The frequent combination of drunkenness with filthiness of personal habits is another circumstance which complicates this question very materially, and renders the appreciation of the value of each a matter of some difficulty. The following table shows the proportion of temperate and intemperate individuals, that were admitted into the Glasgow Fever Hospital from November 1st, 1838, to November 1st, 1839, whose habits could be ascertained with more or less certainty; and the eruptive cases are only included.

| | Temperate. | A little Intemperate. | Intemperate. |
|---|---|---|---|
| E. Typhus (MALES) | 125 | 51 | 73 |
| E. Typhus (FEMALES) | 76 | 8 | 30 |

In this table the proportion of intemperate males is much greater than that of the females. Can this circumstance account, to a greater or less extent, for the greater mortality of the former in almost all hospitals? It would be natural for a person, who wished a certain theory supported, to conclude that as such a large number of those affected with fever were reported to be more or less intemperate, this could not be an accidental and uninfluential concatenation; but that the two circumstances must stand to one another, in the relation of cause and effect. It would be necessary, however, before such an inference could be drawn, to ascertain whether the proportionate amount of the intemperate to the sober was greater in the cases of fever than what existed among the community from whom they were sent. We fear that this question cannot be determined; for the prevalence of intemperance among the working population of large cities has been calculated principally from the amount of drunkards that appear on our streets, from the large and increasing number of our spirit shops, and from the enormous quantity of ardent spirits consumed in a year. And though there can be no doubt that drunkenness has increased among the lower classes to a lamentable extent, its numerical amount has never been ascertained, and perhaps never can be accurately ascertained; but certainly there are grounds for believing that the proportion enumerated as intemperate, in the table which has been given, is not greater than what really exists among the inferior grades of our working population. A similar opinion is entertained by Chomel, who states that alcoholic excesses appear to exert no influence on the production of the typhoid fever. Intemperance, however, tends indirectly to predispose the system to contagion, by the production of filthy habits. It also exercises a most powerful influence in increasing the mortality from fever. In the Glasgow Fever Hospital there occurred eighty-one deaths from eruptive typhus in individuals whose habits were ascertained, and thirty-four of these were reported as intemperate, nineteen a little intemperate, and twenty-eight temperate. In Dr. Craigie's table of the deaths in thirty-one fever cases that occurred in the Edinburgh Royal Infirmary, there are fifteen stated to be irregular or dissipated, only two regular, the habits of the remainder are not stated.*

It is also a singular fact, which has been noticed by several writers, that fever is more fatal among the higher than among the lower classes. Dr. Braken states, in reference to the fever which prevailed at Waterford during the years 1817-18-19, that "it would be difficult to adjust the rates of mortality in the upper classes, but it seems probable that one fourth or perhaps one third of all those persons who were attacked with fever fell victims to its power."†

Drs. Barker and Cheyne, in their historical account of the Irish epi-

* Edinburgh Medical and Surgical Journal, vol. xlvii. p. 296.

† Barker and Cheyne on Fever, vol. i. p. 277.

demic, state that "in every part of the country fever was reported to have been much more fatal amongst the upper than the lower classes."* To what is this difference of mortality, so generally remarked by experienced hospital physicians, to be attributed? and which in Ireland seemed to be very remarkable, namely, in the lower classes about one in twenty-three cases, and among the upper classes one in three or four generally, but in other places about one in seven. Can the difference in the mode of living account for this anomaly? as the first live very much on potatoes, while the other use a larger or smaller proportion of animal food; and the lower classes almost everywhere in this country use less animal food and stimulating dishes than those who are more wealthy and in a higher sphere of society.

This subject is highly worthy of farther investigation; for the difference of mortality which exists among these different classes most probably depends more upon some cause connected with their habits and kind of aliment than upon their *morale*.

*Influence of age.* Almost all modern authors who have written on fever statistically state that the susceptibility to this disease is greater among young persons than among the old; and there is sufficient evidence brought forward to establish this; but certainly the conclusions which have been drawn respecting the greater liability of one period of youth when compared with another have not been satisfactorily proved.

From an examination of the ages of 117 patients, and by comparing his table with the results obtained by M. Louis and some other observers, M. Chomel thinks it may be established that the most common period of life for attacks of typhoid fever is from the eighteenth to the thirtieth year; that it is rarely observed beyond forty years; and that perhaps no case has yet been observed where the patient was beyond fifty-five years.†

Dr. Cowan states that, "from an examination of these (his) tables, it appears that the period of life at which fever is most liable to occur is from the age of twenty to twenty-five years for the males, when the proportion is 21·23 per cent., and from the age of fifteen to twenty for females, when the proportion is 23·83 per cent."‡ Dr. Geary, in his report of the Limerick Fever Hospital states, "that children are much more liable to fever than is generally supposed, and to the little apprehensiveness of disease being transmitted by them may be attributed the spread of disease through families in many instances. It will be seen underneath that nearly one sixth of the admissions for 1836 were under ten years of age, a fact which bears out what we have stated, and is also a satisfactory proof of the increasing confidence which public hospitals are acquiring from the community. . . . . . Of the entire treated for the year, full two thirds were under twenty years of age.§ We have selected the statistics of these two last-mentioned authors chiefly on account of the large number of cases from which their conclusions have been drawn—the first having treated 2257, and the second 3227—in order to show the fallacy of the principles by which the susceptibilities

* Barker and Cheyne on Fever, vol. i. p. 95.
† Chomel, Clinique Médicale, vol. i. p. 311.
‡ Cowan's Vital Statistics of Glasgow, p. 20.
§ Dublin Journal of Medical Science, vol. xii. pp. 98-9.

of persons to fever at the various periods of life are estimated. It is obvious that the proportionate number of cases at the various ages given by the above authors is only that which exists in an hospital; but it by no means follows that the same ratio will be maintained among the general community.

Before any such inference could be drawn, evidence must be brought forward to prove that the admissions of cases into hospitals were in the same proportion as to ages as that which existed among the population from whom they were sent; for it is well known that children in many towns are not so frequently sent to hospitals as adults. And this circumstance may perhaps account for the discrepancy which exists between the conclusions of Dr. Cowan and those of Dr. Geary. This method, however, even though it were ascertained that the same proportionate number of cases affected with fever was admitted into hospitals at the various ages, is very unsatisfactory, as has been pointed out by Dr. Arthur Thomson; for it does not show the number of persons living at each period of life, so that an estimate may be formed of the proportion which the number living at each term of life bears to those who have been attacked. In order to supply this deficiency, the author we have already quoted gives the following table:

"Table IV. *showing the estimated number in the inhabitants at Glasgow at each age during the year* 1836; *the number attacked by fever, together with the ratio attacked out of every thousand at each decennial period of life.*

| Ages. | No. of inhabitants at each age. | No. attacked by fever. | Ratio per 1000 attacked by fever. |
|---|---|---|---|
| Under 10 | 67·469 | 3811 | 56 |
| 10 to 20 | 50·009 | 1539 | 30 |
| 20 to 30 | 46·275 | 1611 | 34 |
| 30 to 40 | 32·044 | 911 | 28 |
| 40 to 50 | 21·758 | 392 | 17 |
| 50 to 60 | 14·090 | 294 | 20 |

" It appears from this table that the greatest susceptibility to fever occurred under ten years of age, after which fever occurs most frequently among persons between the age of twenty and thirty. The number attacked after the age of thirty decreases gradually as life advances."*

This method of calculating the susceptibilities to fever is certainly superior to that which is deduced from the admissions into hospitals; but it is attended with the following objections, which must tend to lessen the accuracy of the conclusions:

1st. The number of fever cases stated in the table is not the result of actual observation, but is calculated from the rate of mortality which occurred at the various terms of life in a fever hospital, on the same principle that Dr. Cowan endeavoured to ascertain the amount of fever in Glasgow, and which is explained at page 62; consequently, the deduction is only an approximation to the truth.

2d. The diseases of which persons die in Glasgow are reported by their friends and not by their medical attendants; and though we acknowledge

* Edinburgh Medical and Surgical Journal, July, 1838, p. 92.

the great value and utility of the mortality bills, even upon this imperfect plan, certainly errors respecting diseases which are sometimes difficult to distinguish from others must frequently take place. This is particularly the case with fever in childhood; which is not so easily recognized as smallpox, measles, and scarlet fever, and which is frequently confounded with hydrocephalus, teething, derangements of the chylopoietic viscera, bronchitis, &c.

3d. If it be admitted that typhus does not frequently attack individuals more than once in their lives, or even upon the principle of its protecting them only for a certain number of years, it follows that there must be a greater number secured by a previous attack among those at the more advanced periods of life than among those who are young. This point has not been prominently alluded to, so far as we are aware, in any previous account of the disease; but in calculating the susceptibilities of persons to fever, those who have previously undergone the disease, or at least a portion of them, ought to be deducted from the general population.* This subject must, therefore, be considered as not thoroughly investigated; and perhaps will remain so until there be some legislative enactment compelling medical practitioners to make a return of all the diseases which have been treated by them throughout the year.

The observations of the British and Irish physicians do not agree with those of M. Chomel, as to the maximum and minimum period of life, beyond which persons are not susceptible of typhus or the typhoid fever. The last-mentioned author thinks it very rarely occurs below ten years of age, and that perhaps no case has occurred where the patient was beyond fifty-five years. Into the Glasgow Fever Hospital there were admitted, during the year 1836, 2257 cases of fever; and out of this number there were 41 under five years of age, and 3 between seventy and seventy-five years.† Into the Limerick Fever Hospital, during the year 1836, there were admitted 3227 cases of fever, and there were 81 below five years of age and 10 between sixty-five and seventy years.‡ Dr. Craigie treated in the Edinburgh Royal Infirmary 7 cases of fever between sixty and seventy years, among 343 admissions.§

We have met with, in the Glasgow Fever Hospital, 5 cases of eruptive typhus in children reported to be three years of age, from the 1st May to 1st November, 1839.

*Acclimatization.* M. Chomel and some other French authors state that the typhoid fever attacks most readily those who have been only a short time in Paris, while those who are natives of that city are more frequently exempted. He mentions that among 92 individuals, 64, that is to say more than two thirds, had lived in Paris, less than two years, while 2 only were natives and residents. The small number of those who were born and resided in Paris is certainly remarkable; at the same time it must be kept in mind that no patient was admitted into his wards below fifteen years of age.

We have constructed the following table in order to illustrate this part

* Dr. Cowan calculates that about 38,000 persons were affected with fevers in Glasgow during the years 1835, 1836, 1837.

† Cowan's Vital Siatistics of Glasgow, p. 20.

‡ Dublin Journal of Medical Science, vol. xii. p. 99.

§ Edinburgh Medical and Surgical Journal, vol. xlvi. p. 35, and vol. xlvii. p. 329.

of the subject; and it comprehends 568 eruptive cases, which were admitted into the Glasgow Fever Hospital from November 1st, 1838, to November 1st, 1839. It shows the number of patients born in Glasgow, the number of strangers, and the duration of their residence in Glasgow.

| | Males. | Females. | Total. |
|---|---|---|---|
| Natives of Glasgow . . . | 77 | 99 | 176 |
| Strangers resident from 1 to 14 days | 12 | 4 | 16 |
| 2 weeks to 1 month . . | 7 | 6 | 13 |
| 1 to 2 months . . . . | 10 | 14 | 24 |
| 2 to 3 months . . . | 10 | 8 | 18 |
| 3 to 4 months . . . . | 5 | 5 | 10 |
| 4 to 5 months . . . | 5 | 3 | 8 |
| 5 to 6 months . . . . | 9 | 12 | 21 |
| 6 months to 1 year . . | 29 | 26 | 55 |
| 1 to 2 years . . . . | 24 | 17 | 41 |
| 2 to 3 years . . . . | 13 | 10 | 23 |
| 3 to 4 years . . . . | 6 | 11 | 17 |
| 4 to 5 years . . . . | 12 | 4 | 16 |
| 5 to 10 years . . . . | 29 | 32 | 61 |
| 10 to 20 years and upwards . | 36 | 33 | 69 |
| | 284 | 284 | 568 |

It appears from this table that among 568 eruptive cases of typhus, in whom this point was ascertained, 176 were natives of Glasgow, and 392 were strangers: 206 of these strangers had resided in Glasgow only from one day to two years, and 186 from two to twenty years and upwards. The strangers amount to about 69 per cent. of the whole number of cases; and those who were affected within two years of their residence in Glasgow to about 52 per cent. of the whole number of strangers.

The following deductions may be drawn from these facts: 1. That strangers are more liable to become infected with typhus fever than native residents. 2. That the majority of strangers are infected within a comparatively short period of their residence in Glasgow. 3. That a minor proportion of the strangers, like the natives of Glasgow, may escape infection for many years, and yet be afterwards attacked. These results support the views which we have elsewhere given of the laws of typhus.

Most of the strangers come from country districts, in which it may be fairly presumed that typhus does not constantly exist, as it does in large towns; it is therefore probable that the majority of them are unprotected by any previous attack; for if typhus attack an individual many times during his life, why should the natives of a town containing 263,000 inhabitants, who are constantly within the sphere of contagion, bear so small a proportion to the strangers.

The facts connected with the propagation of smallpox in Glasgow are of a very similar kind; for the majority of the unvaccinated persons who are sent to the Fever Hospital are Highlanders, who have come very recently from a district where this disease is not in operation, and who consequently have not previously been exposed to contagion.

*Influence of trade or occupation.* Little is known accurately as to the operation of the different trades, in increasing or diminishing the susceptibility to fever. In manufacturing towns there are a greater number of persons connected with cotton manufactures affected with fever than other operatives; but this may be expected; because they generally in such places constitute the most numerous class among the general population. Again, in other towns, labourers are the most numerous class who are affected with fever. Dr. Geary, Physician to the Limerick Hospital, states that "we have a tabular view before us, which shows the number in families of each class of 2416 persons admitted from the city parishes, and the proportion they bear to each other; though the exact relation to the general population cannot be determined, as there is considerable difficulty in ascertaining the amount of each trade. However, as may be expected, the labouring class being the most numerous, constitute the largest number, averaging one half of the entire; and including all, we find that more than one half of those treated for the year cannot be said to be of any trade, namely, females and children."*

It is an ancient opinion that tallow-chandlers, butchers, tanners, and water-carriers are rarely affected with plague or fever. Dr. Hancock quotes the following evidence in reference to the trades that were exempted from the plague. "Volney tells us that at Cairo it is observed that water-carriers, continually wet with the fresh water they carry in skins upon their backs, are never subject to the plague. This fact coincides with the observations in London. George Baldwin, consul-general in Egypt, says that among upwards of a million of inhabitants carried off by the plague in Upper and Lower Egypt, during four years, he could not learn that a single oilman or dealer in oil had suffered. Jackson, in his reflections on the commerce of the Mediterranean, likewise informs us, that in the kingdom of Tunis, there never was known an instance of any of the coolies or porters who work in the oil stores being in the least affected by the disorder; their bodies being always well smeared with oil, as well as their clothes being imbued with it. We are told by Fonseca, that all the tanners at Rome escaped the plague; and Mindererus and Schenck make a similar observation. Dr. Maclean refers to the exemption of tanners at Cairo."† Dr. Tweedie notices the exemption of butchers from fever, and states that though almost every description of mechanics was admitted during the year into the London Fever Hospital, he did not recollect of a single instance of a butcher.‡ Other physicians, however, have met with patients who followed this occupation. Dr. Southwood Smith, in his table of the occupations of 679 patients affected with fever, enumerates three butchers, two curriers, and two skinners.§ Dr. Craigie, in his table of 181 cases of fever treated in

* Dublin Journal of Medical Science, vol. xii. p. 103.
† Hancock on Pestilence, p. 184.
‡ Tweedie's Clinical Illustrations of Fever, p. 79.
§ Southwood Smith's Treatise on Fever, p. 431.

the Edinburgh Royal Infirmary, mentions three butchers among that number.*

The following tables show the various trades, occupations, &c. of 586 patients admitted into the Glasow Fever Hospital from November 1st, 1838, to November 1st, 1839. They include all the eruptive cases of typhus in which the occupation, &c. were ascertained.

Males.

| | | | | | |
|---|---|---|---|---|---|
| Bricklayer | 1 | Fisherman | 1 | Plasterer | 1 |
| Brushmaker | 1 | French-polisher | 1 | Pensioner | 1 |
| Brickmakers | 2 | Glass-cutters | 3 | Printers | 2 |
| Blacksmiths | 9 | Glass-blowers | 3 | Quill-dresser | 1 |
| Bakers | 4 | Gasmaker | 1 | Quarriers | 2 |
| Currier | 1 | Gardener | 1 | Ropemaker | 1 |
| Confectioner | 1 | Ham-curer | 1 | Schoolmaster | 1 |
| Collier | 1 | Hawkers | 5 | Lawyer | 1 |
| Cooper | 1 | Joiners | 6 | Showman | 1 |
| Cabinet-makers | 3 | Labourers | 76 | Shoemakers | 11 |
| Carters | 4 | Last-maker | 1 | Sailors | 6 |
| Carpenters | 3 | Malsters | 3 | Factory-workers | 22 |
| Candle-maker | 1 | Masons | 6 | Servants | 4 |
| Clerks | 2 | Milk-dealer | 1 | Slaters | 3 |
| Coffee-roaster | 1 | Optician | 1 | Tailors | 7 |
| Dyers | 3 | Nailers | 4 | Tinsmith | 1 |
| Engineers | 7 | Policeman | 1 | Turner | 1 |
| Engineman | 1 | Porters | 4 | Tobacconist | 1 |
| Firemen | 3 | Painters | 3 | Wireworkers | 2 |
| Founders | 4 | Potters | 3 | Weavers | 63 |
| | | Watchman | 1 | Warehouseman | 1 |
| | 53 | + | 126 | + | 133 = 312 tot. |

Females.

| | | | | | |
|---|---|---|---|---|---|
| Weavers | 11 | Servants | 38 | Hawkers | 6 |
| Factory-workers | 77 | Fruit-dealers | 2 | Bark-peeler | 1 |
| Sewers | 25 | Washerwomen | 2 | Stocking-knitter | 1 |
| Beggar | 1 | Winders of Yarn | 3 | Straw hat-maker | 1 |
| Shearers | 3 | Calico-printers | 2 | | |
| Married | 97 | Nurses in F. Hosp. | 4 | | |
| | 214 | + | 51 | + | 9 = 274 |

Total of Males and Females = 586

*Influence of Pregnancy.* Among 172 females admitted from May 1st to November 1st, 1839, there were fourteen pregnant, being about 8 per cent. of the whole, and fully three fourths of this number had abortion or premature labour during the course of the disease. This appears a considerable number; but in the present state of our knowledge respecting this point, we are only entitled to conclude from it that pregnancy is not an operative circumstance in preventing the communication of typhus, and this opinion is corroborated by the *general experience* of practitioners. Unless there existed a correct enumeration of the number of individuals belonging to each occupation in Glasgow, no parti-

* Edinb. Med. and Surgical Journal, vol. xlvii. p. 286.

cular deduction could be drawn from these tables; but certainly it is worthy of remark that there should be no butcher,* no tanner, only one currier, only six masons, and one bricklayer, who together are a very numerous class of operatives in Glasgow, while there are seventy-six labourers. The latter class of operatives are generally filthy in their habits and live in small ill-ventilated houses, while masons are comparatively cleanly and comfortable in their circumstances.

The evidence which exists on this point, as has been already stated, is still very imperfect and inconclusive; but certainly butchers and tallow-chandlers or candle-makers appear to be more rarely inmates of a fever-hospital than persons belonging to other trades and occupations who are as numerous in the general population. But there are several circumstances which influence the admissions into hospitals, which ought to be taken into consideration before any conclusion can be drawn from them. 1st. Those operatives who are in better circumstances than the average class of them, with the exception of servants, are more rarely sent to an hospital. 2d. There may exist prejudices in a particular class of operatives against hospitals. Whether any of these objections may apply to the butcher or the candle-maker we are unable with certainty to determine, but undoubtedly the persons who followed these two occupations are not below the average in point of comfort in their circumstances.

M. Parent-Duchatelet has made some very curious and important experiments respecting the absorption of putrid emanations by various substances, which may, by analogy, be made to bear upon this subject. He found that distilled water and soups possessed, in a high degree, the property of impregnation with putrid effluvia; but that greasy bodies covering the surface of the liquid oppose an obstacle to the passage of these emanations. The following is his eighteenth experiment: "It might be useful to know if there were any means capable of preventing liquids from being impregnated with putrid emanations; this means chance furnished me with. Having set aside a certain quantity of *bouillon* as an experiment, I found it next day covered with a pellicle of grease, and below this grease it was in a most natural state; inferring from this experiment I poured two or three drops of oil into each of the experimental dishes filled with *bouillon*, as well as into the others filled with water, and after they had remained twenty-four hours among the putrid emanations I remarked that none of these liquids had contracted odour, but the surface of oil gave out in all the cases a very powerful odour."† Solid substances were also infected with the odour of putrid emanations, such as beef and wood,‡ and water, completely inclosed in a piece of intestine, bladder, or strong parchment, was even tainted with it.§ He ascertained also that camphor, valerian, and mineral tar communicated their odour to water when it is exposed to the effluvia arising from these substances.||

Although it has not been demonstrated experimentally, it seems highly probable that contagious effluvia, like fetid emanations, are soluble

* One patient had been a butcher, but had worked as a labourer for six months before he was affected.

† Annales d'Hygiène Publique, tom. v. p. 39.

‡ Ibid., p. 44. § Ibid., p. 39. || Ibid., p. 38.

in water, from the fact that thorough ablution of the clothes of persons who have laboured under fever disinfects them completely. Hence the advantage, as a prophylactic, of frequently sponging the skin of a typhus patient with water, more especially as tepid sponging is useful in the treatment of the disease. It appears, also, that contagious effluvia are volatile, like the emanations from putrid bodies, and may be separated from substances to which they adhere by means of heat. The late Dr. Henry of Manchester found that clothes impregnated with the miasmata of scarlatina and typhus were disinfected by exposing them to a temperature of 204° F. for one hour and three quarters, and that they did not induce any of these diseases when afterwards worn by healthy individuals.*

Are we then entitled to believe that butchers, candle-makers, &c. are more rarely affected with fever than other operatives? Dr. Tweedie supposes the exemption of butchers to depend on their good living; but it appears to us that the common theory respecting the operation of oily or greasy bodies in preventing fever will also explain the matter, and will apply to the butcher as well as to the tallow-chandler. It has already been shown by the experiments of Parent-Duchatelet, that greasy bodies attract powerfully putrid emanations; and it is well known that they unite very readily with odoriferous bodies of almost every kind; is it not therefore probable that contagious effluvia are regulated by a similar attraction, more especially when this hypothesis is coupled with the commonly received opinion in eastern countries, that oil is a prophylactic to contagion. If this be granted, how then does an oily or greasy body protect the butcher or the candle-maker? In the exercise of their various manipulations, the persons belonging to these two occupations have their clothes and the uncovered parts of their bodies more or less imbued with grease, an accompaniment which they almost constantly carry about with them. The contagious effluvia may, therefore, in place of being absorbed by the skin, combine permanently with the fatty body, and in this be fixed and rendered harmless.

We only bring forward this as an hypothesis capable of accounting for the generally received opinion respecting the protecting property of oil; but certainly if there be prophylactic powers in it or in any other substance, it is well worthy of being investigated experimentally.

* Philosophical Magazine, Nov. 1831.

### *On the Identity of Typhus and the Typhoid Fever.*

As we have made several quotations from M. Chomel, as well as from M. Louis, who seem to think that the typhoid fever of France is a different disease from the ordinary British typhus, it may be necessary to show, although it may appear foreign to this essay, upon what grounds we consider them identical. The evidence by which the identity of typhus and the typhoid fever may be established, consists of two kinds, namely, the symptoms during life, and the morbid appearances after death; and in order that the subject may not be entrammelled with unnecessary detail, those symptoms and lesions only which in the aggregate are reckoned diagnostic of the disease shall be described. M. Chomel describes the disease under three septennary periods, each being characterized by peculiar symptoms. First period is characterized by feebleness, stupor, sleeplessness, mutterings, meteorismus, diarrhœa, sensibility of the abdomen, and a sense of fluid gurgling in the lower half of the belly, epistaxis, the typhoid eruption, and frequent pulse. Second period is characterized by the eruption which M. Chomel admits to be similar to that described by Hildenbrand, as observed in the *typhus castrensis*, sudamina, ulcerations and sloughs on various parts, chops and ulcers in the tongue, increased stupor, unconsciousness, dorsal decubitus, difficulty of deglutition, involuntary evacuations, retention of urine, subsultus tendinum, picking of the bedclothes, general and permanent rigidity of the members, deafness, coma, small weak tremulous pulse, or throbbing and intermittent, and varying in frequency from 80 or 90 beats to 120 in a minute, but which sometimes sinks to 40 or 50, a fuliginous coating of the tongue, teeth, gums and lips, diarrhœa, intestinal hemorrhages, increased meteorismus, respiration more constrained, fetid exhalations from the skin and breath. Third period. It is generally during this stage that the febrile disorder subsides, whether the patient recovers or dies. When the termination is going to be favorable the patient becomes more sensible, is more disposed to sleep, the mouth and tongue become more moist, the fecal discharges more natural, and the pulse becomes less frequent. On the other hand, when the termination is going to be unfavorable, the stupor increases, there is an alteration in the features, stertorous breathing, feebleness of pulse, a drier skin, or cold and covered with clammy sweat, general emaciation, hollow eyes, tremulous speech, indistinct and murmuring answers to questions, extreme feebleness, coma, and death. Sometimes death is accelerated by the occurrence of tetanic or epileptic paroxysms, and intestinal perforations and erysipelas are mentioned as occurring during convalescence. Any practitioner who has paid close attention to the symptoms of British typhus will readily discover their identity with those so well described by M. Chomel, as indicating the typhoid fever. There are, however, too or three symptoms which he places more dependence upon as characteristics of the disease than what is generally done in Britain, which it is necessary to notice more particularly. He represents diarrhœa as a very common symptom in the majority of cases, there being from four to eight alvine evacuations daily.

Now this symptom by no means occurs frequently in Britain, but this discrepancy may, to a certain extent, be explained, for the French physicians seldom exhibit purgatives in case of aggravating the *gastro-enterite;* hence the solid excrementitious matter which naturally accumulates in the torpid bowels of a typhoid patient will produce a morbid secretion from their excited surfaces, and being tinged with feculent matter may represent a fecal diarrhœa. This view is supported by the admission of M. Chomel himself. He states that "in some cases, at the time when the first improvement in the symptoms occurs, the alvine evacuations consist of firm, figured motions, to the great astonishment of the attendants, who with difficulty understand how such a change could be effected in so short a time. It is probable that these matters had remained during the whole period of the disease in some of the cells of the colon, and had not prevented the passage of liquid motions. There are discharged sometimes in these cases prodigious quantities of black dry matters."* From M. Chomel's account it would appear that meteorism or tympanitic swelling of the belly is more frequent in France than in Britain, for it has never been considered in this country as peculiarly characteristic of typhus. This discrepancy may, however, be reconciled, for according to this author the meteorism is only to be discovered in the early stages by percussion, while in the latter stages it is discoverable from the convex form of the belly. British practitioners apply the term tympanitis only to prominent distention of the belly by flatus, while those in France apply it not only to this but to minor enlargements not discoverable by the eye. Epistaxis is another symptom which M. Chomel considers frequent, and of great value as a diagnostic of typhus, especially if it occur during the first days of the disease. These hemorrhages are not profuse, but are most generally only a few drops, either from the anterior part of the nasal cavities or from the posterior by the throat, in the form of mucous masses, streaked and mixed with blood. Bleeding from the nose or mouth is certainly not so frequent in Britain as to constitute a diagnostic symptom of typhus, although it does occasionally occur; but it is generally hemorrhage to a considerable extent which has been noticed by authors in this country, and we do not doubt that the smaller discharges of blood or bloody mucosities have occasionally been overlooked or not attended to as unimportant. M. Chomel, although he does not appear to be prefectly convinced that typhus and typhoid fever are the same disease, is strongly inclined to this opinion from the similarity of their symptoms. He says that "another point which is still in favour of the opinion of contagion is the analogy which exists between the typhoid affection and typhus of camps, the contagious character of which is contested by no person. If we compare these two diseases, and from our recollections and from the description which has been given by Hildenbrand, and which it was in our power during 1814 to verify the accuracy, we shall find the same symptoms in the two affections, both of them commence by headach, with most subjects prostration and stupor appear at the beginning, and not solely, as in other affections, after the malady has endured a long time, and has very greatly debilitated the organism. The other symptoms, such as the meteorism, the diarrhœa,

* Chomel, Leçons de Clinique Médicale, tom. i. p. 42.

the notable weakness of the senses, the tendency to ulcerations and hemorrhages are common to the two diseases. The progress is the same in the two diseases, inflammatory symptoms predominate at first and are afterwards followed by nervous or adynamic phenomena. One of the few differences which we have observed between these two affections consists in the duration, which is more prolonged in the typhoid affection than in typhus. This last ceases generally about the fourteenth day, whilst it is rare that the first terminates before the twentieth day. Another difference consists in the frequency with which true petechiæ or purple spots are observed in typhus, which are comparatively rare in the typhoid malady. With regard to the cutaneous exantheme or typhoid eruption, it presents the same characters in the two affections; the only differences are in the number of spots and in the period of their appearance. In place of being confined, as they are most frequently in the typhoid fever, to the belly and chest, the lenticular spots in typhus cover and in greater numbers almost the whole surface of the body. In this last the eruption is developed generally about the fourth day of the disease; in the typhoid fever it appears only about the eighth day, and sometimes much later. . . . . The only difference which Hildenbrand and Pringle admit between typhus and the most of other fevers which we have referred to the typhoid malady is that the severity of the disease is greater in typhus, its progress more rapid, the adynamic phenomena more decided, and the eruption more general; but these differences are not sufficient to make us reject the identity of the malady, for they may depend upon circumstances more or less troublesome, during which it is propagated. These differences may rather indicate degrees of intensity than that they are maladies entirely distinct."*

These distinctions between typhus and typhoid fever, as stated by M. Chomel, must appear to every one sufficiently acquainted with the typhus of Britain as very unimportant, for in young persons the eruption is frequently observed upon the extremities as well as upon the breast and belly, and even in the same family, when the disease ought to be acknowledged as identical, the number of spots observed on each member of it often varies exceedingly. It is also a well-known fact that complete convalescence from typhus fever rarely takes place on the fourteenth day except in young persons; while among those more advanced twenty or a greater number of days may elapse before this occurs.

In order to show still further the identity of the symptoms of typhus with the typhoid fever, we shall quote the observations of a very accurate and experienced physician, Dr. Lombard, of Geneva. He states that "with this experience and having witnessed numerous dissections of subjects dead of typhus fever, and having found in every one of them at Paris and at Geneva the morbid state of the intestinal canal which the French pathologists consider as essential; under these circumstances, when I arrived in Great Britain and had an opportunity of seeing the fever cases here, and when I found that they presented a very great similarity, if not an identity, of symptoms with those I had been for years in the habit of observing, it is not to be wondered at, I say, that I should have expected to find exactly the same post-mortem appearances.

* Chomel, Leçons de Clinique Médicale, tom. i. p. 335.

I mentioned this subject to my friends at Glasgow, and they allowed me to dissect the body of a person in whom I said no doubt could exist as to the presence of follicular disease; judge then, how great was my astonishment at not being able to detect a single trace of this morbid change in any part of the intestinal canal, and at finding no marks of disease save some redness and softness of the mucous membrane of the stomach, which may have been produced by inflammation, but more probably was owing to muscular congestion, occurring during the last stage of the disease, or even during the agony that precedes death."*

Dr. Lombard, however, was not convinced by this inspection; and on his arrival at Dublin he examined the bodies of two patients who had died of typhus at different hospitals, and with the same results. It thus appears that the symptoms of typhus and the typhoid fever are nearly the same, and that they cannot be distinguished from one another; so that upon this ground their separation cannot be maintained. But those who support the difference of the two affections rest their proof chiefly upon the pathological lesions which are found in the intestines.

M. Louis characterizes the typhoid fever under the following description: "An acute malady accompanied with a febrile movement more or less intense, variable in its duration, proper to young persons, chiefly to those who are placed within a short time in circumstances new to them, the cause of which is unknown, commencing by a violent shivering, anorexia, thirst, and in the great majority of cases by colics and diarrhœa, very soon accompanied by feebleness which is small in proportion to the other symptoms, then more or less quickly somnolence, stupor, delirium, meteorismus, sudamina, lenticular rose-coloured spots, ulcers on the sacrum, ulcerations more or less deep of the skin, in the parts occupied by blisters, deafness, various spasmodic movements, or permanent contraction of the limbs; symptoms some of which disappear after a certain time, others increase for the most part in a progressive manner, when the patients die, or diminish more or less rapidly, at length to disappear altogether if the affection has a happy termination; the anatomical characters of which consists in a special alteration of the elliptic plates of the ilium.† . . . . Of all these lesions one only is constant, being found in all the subjects: I speak of the alteration of the elliptic plates of the small intestines, to which may be added the alteration of the mesenteric glands; I have regarded it as inseparable from the existence of the affection under review in forming the anatomical character. And as it was more or less great with some subjects who died on the eighth day of the disease, as with the greatest number the first symptoms indicated a lesion of the intestinal canal, as the alterations of the small intestines was greater than those of the colon, which was sound in a sufficiently large number of cases, I am warranted to conclude that the alteration of the elliptical plates commenced at the beginning of the disease."‡ M. Chomel, although he appears strongly inclined to support the doctrines of M. Louis and the other French pathologists, makes the following candid avowal of his opinion deduced from a rigid examination of all the pathological facts connected with the typhoid fever: "If, to this consideration furnished by analogy, we join these two other

* Dublin Journal of Medical Science, vol. x. p. 18.

† Louis de Gastro-Entérite, tom. ii. p. 317.

‡ Ibid., tom. i. p. 449.

circumstances already established: 1st, that there is no constant proportion between the severity of the symptoms and that of the lesions of the follicles; 2d, that the lesion has been completely absent in subjects who had offered during life all the symptoms of typhoid affection—it will become still more evident that the typhoid malady does not consist essentially of inflammation of the follicles; that this inflammation is only one of the phenomena of the disease, that it belongs, like most of the disseminated inflammations, to secondary inflammations; that it may be compared as to its pathogenic power not even to the pustules in variola, for in this there is always a proportion between the number of the pustules and the severity of the malady, but rather to the bubo in the pestilence of the East."*

M. de Claubry, in his prize essay read before the Royal Academy of Medicine, has adduced very copious evidence to prove the identity of typhus and the typhoid fever. He controverts the opinions of M. Louis respecting the ages that are exempt from the typhoid fever, and states that "it is not rare to see the disease in the Parisian hospitals at the age of four, six, eight, and ten years; and that M. Andral has witnessed it after seventy years."† He adduces Fauvages, Reveillé, Parise, Thruvenel, Ducastaing, and Pellerin to prove that ulcerations having elevated borders and exposing the peritoneal coat were found near the extremity of the small intestine in typhus.‡ The same author also shows that the typhoid fever spreads by contagion in the same way and under the same circumstances as typhus.§ Dr. Lombard, who contends for the distinction of the two diseases, adduces similar evidence to prove that typhoid fever is possessed of contagious qualities.||

We think it unnecessary to adduce evidence to prove that the follicular disease of the intestines is greatly less frequent in British typhus than in the continental typhoid fever; for the pathological investigations which have been made in England, Scotland, and Ireland regarding this point are now numerous and well known. Indeed in this country, in place of finding in almost every subject who died of typhus fever disease in the agminated or solitary glands, the minority has been the proportion found in many hospitals, and the affection of the spleen and brain more frequent than that of the intestines. If then there be no specific difference between typhus and typhoid fever; why are the pathological lesions of the intestines so much more common and intense in France than in Britain? It is perhaps not possible to give a satisfactory answer to this question, unless a difference of climate, diet, habits, &c. be allowed a certain influence. Dr. Lombard, in his first letter to Dr. Graves, seemed to have formed a very correct opinion respecting the nature of typhus, although he afterwards thought proper to change his views. In his first letter he says that "all these considerations, my dear friend, seem inevitably to lead to the conclusion that typhus fever is more a general disease affecting the whole constitution than a malady depending on a local inflammation or any local change of structure. May we not infer, also, that various causes serve to impress

* Chomel, Leçons de Clinique Médicale, tom. i. p. 536.
† Mémoires de l'Académie de Médecine, vol. vii. p. 190.
‡ Ibid., p. 80. § Ibid., p. 120.
|| Lombard's Clinical Remarks on Bilious and Typhoid Fevers, p. 17.

upon this general disease a tendency to associate itself with and produce various local ailments; among these causes, the most influential probably are, climate, seasons, the race of mankind, diet, and various circumstances which act powerfully both on the mind and body, and which when concentrated at any one point of time have given rise to those various epidemics of typhus that have so frequently devastated the different countries of Europe."* The same author, however, in his second letter to Dr. Graves, assumes his old hypothesis that the two fevers are different, and goes even a step further, for he maintains that both kinds are to be met with in the British and Irish hospitals. His views seem to be included in the following quotation from his letter: "But the Irish contagious fever is not the only source of typhoid diseases in Great Britain; the sporadic continued fever, observed in all parts of Europe, is also to be found in the different towns of the British empire. This fever, characterized by the follicular intestinal eruption and by consequent ulcerations, is to be seen in the different places above mentioned; in Glasgow it forms one third of the total number of cases;† in Dublin the proportion is much less; in London it is one fourth, and varies in the different seasons, because the continued sporadic fever is much under the influence of the temperature, being more frequent in autumn than in spring and winter; a proof that the proportion of this sporadic fever is the cause of the greater proportion of ulceration cases found at times in the British hospitals, as already mentioned. Having stated my opinion on your British continued fever, I resume it in the following theoretical view: You have two different fevers, one highly contagious, which I may call the Irish typhus, and in which the cephalic symptoms predominate to the exclusion of abdominal alterations; the other which is sporadic and most likely not so infectious, and in which the abdominal symptoms are more predominant, so much so that the follicular disease and consequent ulcerations are always to be found."‡ Dr. Gerhard, of Philadelphia, is another author who endeavours to show that there is a specific difference between typhus and typhoid fever, and that both are to be met with in Philadelphia. He makes the following observations respecting the post-mortem appearances which were observed in the American typhus: "In this large number of autopsies, amounting to about fifty, there was but in one case, and that doubtful in its diagnosis, the slightest deviation from the natural appearance of the glands of Peyer. In the case alluded to, in which there had been some diarrhœa, the agglomerated glands of the small intestines were reddened and a little thickened, but there was no ulceration and no thickening or deposit of yellow puriform matter in the submucous tissues. The disease of the glands resembled that sometimes met with in smallpox, scarlet-fever, or measles, rather than the specific lesion of dothonenteritis."§

* Dublin Journal of Medical Science, vol. x. p. 23.

† In some places in Scotland ucleration of the intestines seems to be very frequent. Dr. John Reid states that Dr. Goodsir, of Anstruther, examined ten bodies, and in every one the elliptical patches of Peyer and the solitary glands at the lower part of the ilium were elevated and ulcerated, and in four, perforation of the intestines had taken place. Edinb. Medical and Surgical Journal. Oct. 1839, p. 459.

‡ Dublin Journal of Medical Science, vol. x. p. 104.

§ American Journal of Medical Sciences. February, 1837.

50

LIBRARY OF THE
Orleans Parish Medical Society

Dr. Gerhard's account of the epidemic typhus in Philadelphia is written with great accuracy, and his post-mortem inspections seem to have been conducted with much care and ability; but his results are certainly not what might be expected from a disease of the same nature as British typhus, which he describes it to be. For though we by no means believe that the lesion of Peyer's glands is a necessary concomitant of typhus, we are certainly supported by British observations when we state that there never were fifty consecutive inspections of typhus subjects made in this country without finding one decided instance of disease in the intestinal follicles.

It is quite evident that Drs. Lombard and Gerhard lay almost the whole weight of the diagnosis of typhus from the typhoid fever, upon the lesions of the intestinal follicles observed in the latter disease; for the almost identity of their symptoms during life are admitted; and is there any British practitioner that could distinguish those cases of eruptive typhus that had diseased follicles from those that had not? Again, it may be asked, what is the peculiar character of the diseased follicles, which constitutes the distinction between typhus and typhoid fever? In subjects dead of typhus fever which we have examined, the follicles are generally found with their margins only distinctly defined, but with little elevation or thickening of the subjacent textures, but such as to give a comparative opacity to the patch; when viewed with a magnifier, their surface presents irregular mammillated projections, bounded by corresponding depressions; sometimes there is only one patch, more frequently two or three, or a large irregular coalescence of patches at the ileo-cæcal valve; deep ulceration is not very common except in protracted cases; and occasionally there is the appearance of superficial ulceration. Now, if the anatomical and distinctive character of the typhoid fever be a morbid alteration of Peyer's glands, one single diseased patch, characterized by its defined margin, greater or less elevation and opacity, ought to constitute the disease as definitely as if there were twenty; just as smallpox is as essentially distinguished by twenty or thirty pustules as by several hundreds. If this be denied, where lies the line of separation?

Does it consist in a certain elevation of the follicles capable of admeasurement, in the deposition of a yellowish white or puriform matter in their subjacent textures, or in a certain amount of ulceration? But it may be argued that there are two species of fever in Britain, the one characterized by a peculiar disease of the intestinal follicles, and the other unaccompanied by any such lesion; and that some slight disease, characterized by a slight elevation and configuration of the patches, does sometimes take place in the latter, such as occurs in scarlet fever, smallpox, &c.; but that this lesion is totally different from that described by Louis and Chomel as characteristic of the typhoid fever. Now, we are ready to admit, at least as far as our experience goes, that the elevation and texture of the follicles are not in many cases precisely similar to those which are stated to be characteristic of the typhoid fever; but certainly they are even in this state morbidly affected.

The following table shows the lesions that appeared on the inspection of 63 eruptive cases, that were admitted into the Glasgow Fever Hospital from 1st May to 1st November, 1839, and it includes both male and female patients in nearly equal proportions:

| | |
|---|---|
| Abnormal serum in brain | 34 |
| Bronchia red | 25 |
| Spleen rather large and soft | 14 |
| Spleen large and pulpy | 30 |
| Peyer's glands enlarged 1 to 3 | 12 |
| Peyer's glands enlarged 3 to 6 | 14 |
| Peyer's glands enlarged 6 and upwards | 22 |
| Solitary glands enlarged | 14 |
| No intestinal glands enlarged | 12 |
| Ulceration of intestines | 13 |

The mesenteric glands were almost uniformly enlarged when ulceration of the intestines was present, but very rarely in other cases.

Now it may be contended that this simple enlargement or figuration of the intestinal follicles is a different affection from that which occurs in the continental typhoid fever, and hence ought to have a different classification. Such an assumption would lead to an endless and very unphilosophical division, and obviously to the formation of three species of typhus, out of the various complications or appearances which are observed in the intestines; namely, 1st, typhus without any intestinal affection whatever; 2d, typhus with simple enlargement of Peyer's glands; 3d, typhoid fever complicated with the follicular affection described by M. Louis; for if one author distinguish a species by a peculiar morbid appearance of the intestinal follicles, another has the same right to form a second, if the affection of these glands, in a certain number of other cases, be denied a pathological similarity to the first; whilst the morbid affections of the spleen, the lungs, the brain, &c. might all be brought forward to increase the subdivision still farther. The strength of our argument, however, that typhus and typhoid fever are the same diseases modified by place, season, epidemic influence, and perhaps by circumstances not yet ascertained, lies in the fact, that it has been admitted that cases of the latter disease, although rare, have occurred without any morbid appearance being discovered in the intestinal follicles; proving that this morbid condition of these glands is not a necessary anatomical character of the disease, such as hepatization or suppuration is of pneumonia, or serum of hydrocephalus. It has also been admitted that the intensity of the symptoms is not proportional to the lesions which ought to occur if the latter were the cause of the former; and it would be contrary to all experience to attribute the formidable symptoms of typhus or the typhoid fever to the lesion of one or two intestinal follicles, even though affected in the form described by the French writers. Would it not, therefore, be refining our classification of diseases beyond all precedent, to separate typhus and typhoid fever into two species, where it has been shown that the symptoms in both are the same, or very nearly so, that they have nearly the same laws, as far as these have been ascertained; that the severity of the symptoms in both is not in proportion to the lesions of the intestinal follicles; and that the other complications of both are similar, although various in the same places at different periods, while the only characteristic in dispute has been acknowledged not a constant and therefore not a necessary element for the existence of the disease.

